# Substantial Epigenetic Variation Causing Flower Color Chimerism in the Ornamental Tree *Prunus mume* Revealed by Single Base Resolution Methylome Detection and Transcriptome Sequencing

**DOI:** 10.3390/ijms19082315

**Published:** 2018-08-07

**Authors:** Kai-Feng Ma, Qi-Xiang Zhang, Tang-Ren Cheng, Xiao-Lan Yan, Hui-Tang Pan, Jia Wang

**Affiliations:** 1 Beijing Key Laboratory of Ornamental Plants Germplasm Innovation & Molecular Breeding, National Engineering Research Center for Floriculture, Beijing Laboratory of Urban and Rural Ecological Environment, Key Laboratory of Genetics and Breeding in Forest Trees and Ornamental Plants of Ministry of Education, School of Landscape Architecture, Beijing Forestry University, Beijing 100083, China; makaifeng@bjfu.edu.cn (K.-F.M.); chengtangren@163.com (T.-R.C.); htpan2000@163.com (H.-T.P.); wangjia8248@163.com (J.W.); 2 Beijing Advanced Innovation Center for Tree Breeding by Molecular Design, Beijing Forestry University, Beijing 100083, China; 3 Mei Research Center of China, Wuhan 430074, China; important12@sina.com

**Keywords:** DNA methylation, flower color chimera, bisulfate sequencing, transcriptome, comparative epigenomes, transposon, ornamental *Prunus mume*

## Abstract

Epigenetic changes caused by methylcytosine modification participate in gene regulation and transposable element (TE) repression, resulting in phenotypic variation. Although the effects of DNA methylation and TE repression on flower, fruit, seed coat, and leaf pigmentation have been investigated, little is known about the relationship between methylation and flower color chimerism. In this study, we used a comparative methylomic–transcriptomic approach to explore the molecular mechanism responsible for chimeric flowers in *Prunus mume* “Danban Tiaozhi”. High-performance liquid chromatography-electrospray ionization mass spectrometry revealed that the variation in white (WT) and red (RT) petal tissues in this species is directly due to the accumulation of anthocyanins, i.e., cyanidin 3,5-*O*-diglucoside, cyanidin 3-*O*-glucoside, and peonidin 3-*O*-glucoside. We next mapped the first-ever generated methylomes of *P*. *mume*, and found that 11.29–14.83% of the genomic cytosine sites were methylated. We also determined that gene expression was negatively correlated with methylcytosine level in general, and uncovered significant epigenetic variation between WT and RT. Furthermore, we detected differentially methylated regions (DMRs) and DMR-related genes between WT and RT, and concluded that many of these genes, including differentially expressed genes (DEGs) and transcription factor genes, are critical participants in the anthocyanin regulatory pathway. Importantly, some of the associated DEGs harbored TE insertions that were also modified by methylcytosine. The above evidence suggest that flower color chimerism in *P*. *mume* is induced by the DNA methylation of critical genes and TEs.

## 1. Introduction

*Prunus mume* Sieb. et Zucc. (2*n* = 2*x* = 16), a well-known ornamental tree, is widely grown for its fruits and its abundant, colorful flowers with their unique fragrance [1]. Following the domestication of this species more than 3000 years ago in China, the cultivation of *P. mume* has spread widely to other countries in East Asia. Its petal color, which ranges from white to pale yellow, pink, red, and reddish-purple, determines the desirability and economic value of individual plants and is one of the central ornamental features attracting viewers and admirers. In the 1940s, varieties with a novel characteristic, flower color chimerism, were discovered and have since served as important materials for landscaping applications and genetic improvement [2]. Drawing on our breeding experience with these varieties, we recognize five types of flowers according to petal color patterns and arrangements on individual trees: (i) bicolored flowers, which are mostly white with some red-spotted or streaked petals; (ii) pure white flowers and (iii) pure red flowers, which are both found together in the same cluster; (iv) white flowers from branches bearing white flowers only; and (v) red flowers from branches bearing red flowers only. Although graft-propagated branches bearing chimeric flowers (i, ii, and iii) produce chimeric individuals, branches with only single-color flowers (iv and v) generate single-color clones. However, at present, little information is available on the genetic mechanisms of *P*. *mume* floral chimerism.

At the cytological level, the formation of plant chimeras is believed to be linked to genetic changes in primordial cells located in the apical meristem that then proliferate mechanically [3]. The resulting somatic cell lines contain pigments, including flavonoid, carotenoid, and betalain secondary metabolites, that can be directly visualized [4,5,6,7]. At the molecular level, chimeric variation was initially explained by the action of transposable elements (TEs, or transposons), or, more specifically, the *Activator*/*Dissociation* (*Ac*/*Ds*) system that regulates the mixture of purple and yellow pigments in maize kernels by activating or repressing a *C* group gene [8]. Much later, the insertions of TEs *Tpn1*, *Tpn2*, and *Tpn3* into structural genes *dihydroflavonolreductase-B* (*DFR-B*), *chalcone isomerase* (*CHI*), and *chalcone synthase-D* (*CHS-D*), respectively, were revealed to be the critical factors giving rise to the corresponding variegated color mutants *flecked*, *specked*, and *r-1* [9,10,11]. The integration of *Tpn4*, an *En*/*Spm*-related transposon, into the *purple-mutable* (*pr-m*) gene encoding a vacuolar Na^+^/H^+^ exchanger was found to be responsible for a mutation giving rise to purple flowers with blue sectors [12,13]. A similar functional mechanism has been linked to tobacco flower color [14]. Other examples include *Gret1* activation regulating the expression of *VvmybA1* to produce colorless grape skin [15]; *Tam1* transposon insertion in *Glycine max* [16]; and the insertion of either *Ty1dic1* or *Retdic1* in *AA5GT*, whose disruption prevents glycosylation at the 5 position of anthocyanins in *Dianthus caryophyllus* [17].

Other studies have uncovered evidence supporting a relationship between chimeric petals and the expression of structural/biosynthetic genes encoding enzymes and other regulatory factors involved in floral pigment biosynthesis and metabolism. For instance, *CHS*, *cinnamate-*4*-hydroxylase* (*C4H*), *flavanone* 3*-hydroxylase* (*F3H*), *DFR*, *anthocyanidin synthase* (*ANS*), and *UDP-glucose*: *flavonoid* 3*-O-glucosyltransferase* (*UFGT*) genes have been found to exhibit differential expression patterns between red and white flower petal tissues of individual higher plants [7,18,19,20,21]. The alternative splicing of *ANS* results in red flower petals [22], while the sequence-specific silencing of *CHS* generates white sectors in *Petunia hybrida* “Red Star” flowers [23]. Flower color variegation was also observed when the *regulator involved in anthocyanin transport* (*Riant*) gene, encoding a GST protein, was expressed while harboring an insertion–deletion polymorphism in exon 3 [24], and a TT2-like R2R3 MYB has been shown to regulate anthocyanin biosynthesis in flowering *P*. *persica* “Genpei” [25]. At the same time, epigenetic modification, such as the use of hypomethylated promoters of *A1*, *DFR*-*B*, and *OgCHS* genes driving the brick-red pelargonidin pigmentation of flower tissue [11,26,27], has been introduced to reveal genetic variation in variegated flowers.

With reference to the previous example, DNA methylation indeed appears to be one of the best-studied epigenetic modifications regulating eukaryotic growth and development [28,29,30,31,32] that also leads to morphological abnormalities in plants [33,34]. For instance, the extensive methylation and transcriptional silencing of a *Lcyc* gene leads to a fundamental change in floral symmetry, from bilateral to radial flowers [35], while methylated genes encoding MYB transcription factors are inversely associated with red and green-skinned fruits of apple and pear cultivars [36,37,38]. Importantly, DNA methylation modification is related to the silencing or reactivation of TEs that generally remain inactive [39,40,41,42]. However, little research has focused on the relationship between methylated TEs and chimeric traits.

In this study, we first assumed that the flower color chimerism of *P. mume* is associated with DNA methylation modification of structural genes or regulators, as well as methylated TEs, through the color regulation pathway. We performed transcriptome sequencing (RNA-seq) and advanced single base resolution methylome detection, which is a technique that has been used to elucidate fruit ripening in tomato [43], dynamic changes during seed development in soybean [44], photoperiodic sensitivity in cotton [45], and drought stress in cotton, apple, and rice [46,47,48], to examine three issues: (i) the methylome landscape of *P. mume*; (ii) differentially methylated region (DMR)-related genes contributing to pigment variation; and (iii) the question of whether TEs with DNA methylation modification contribute to bicolored flower formation.

## 2. Results

### 2.1. Variation in Pigmentation in White (WT) and Red (RT) Petal Tissues

**Figure 1:**
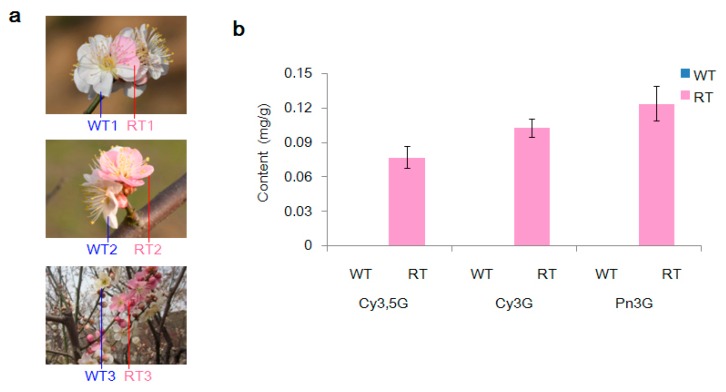
Color phenotypes and petal-tissue anthocyanin contents of flowers of *Prunus mume* “Danban Tiaozhi” collected in this study. (**a**) Examples of the six types of sampled petals. Samples WT1 and RT1 were respectively collected from white and red petals of bicolored flowers; WT2 and RT2 were collected from flowers with only white or red petals, respectively; WT3 and RT3 were collected from flowers on branches with only white flowers and only red flowers, respectively. (**b**) Anthocyanin content (mg/g fresh weight) of white petal tissue (WT) and red petal tissue (RT) samples. Cy3,5G, cyanidin 3,5-*O*-diglucoside; Cy3G, cyanidin 3-*O*-diglucoside; Pn3G, peonidin 3-*O*-glucoside.

Three types of anthocyanins, namely, cyanidin 3,5-*O*-diglucoside (Cy3,5G; 0.077 mg/g fresh weight), cyanidin 3-*O*-glucoside (Cy3G; 0.103 mg/g fresh weight), and peonidin 3-*O*-glucoside (Pn3G; 0.124 mg/g fresh weight), were detected in red petal tissue (RT) samples by high performance liquid chromatography-electrospray ionization-mass spectrometry (HPLC-ESI-MS). In contrast, no compounds similar to these secondary metabolite products were detected at an absorption wavelength of 520 nm in white petal tissue (WT) samples (Figure 1a,b and Figure S1).

### 2.2. Genome Methylation Landscape of Prunus mume

**Figure 2:**
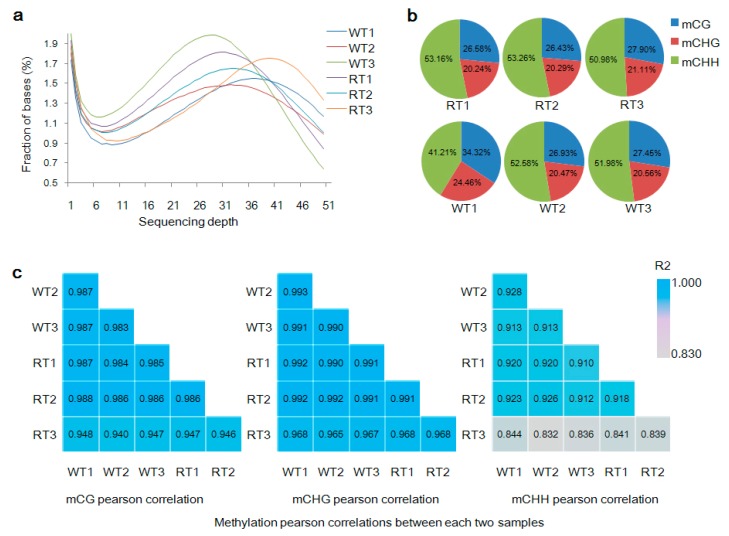
Genome sequencing coverage and methylation percentages and correlations between samples. WT1, WT2, and WT3 represent white petal tissue samples 1, 2, and 3, respectively. RT1, RT2, and RT3 represent red petal tissue samples 1, 2 and 3, respectively. (**a**) Distribution of the coverage of the six sequenced genomes. The *x*-axis represents sequencing depth, and the *y*-axis indicates the percentage of bases covered. (**b**) Percentage of methylated sites by sequence context (mCG, mCHG, and mCHH) relative to total methylated sites within each sample genome. (**c**) Pairwise correlations of methylation levels by sequence context (mCG, mCHG and mCHH) between sequenced genomes.

We used the BS-seq method, the “gold standard” of DNA methylation detection, to reveal the methylomes of six petal tissue groups from a single ornamental tree. In total, 40.0–50.9 million clean reads were generated, which corresponded to 11.6–14.6 Gb and greater than 41-fold coverage of the genome (estimated size = 280 Mb). To confirm the quality of the sequences, we also calculated QC20 (>97%) and QC30 (>92%) values, the bisulfite conversion rate (>99%), and GC content (21.37–21.54%). Approximately 59% of clean reads could be mapped to the reference genome, with a duplication rate of approximately 11.13–19.09% (Table S1). As revealed by sequencing coverage statistics, the maximum coverage was obtained at a sequencing depth of approximately 30× to 40× of the reference genome (Figure 2a), with each chromosome sequenced at a depth of around 24× to 34.2× (Figure S2). The coverage of cytosine sites, 8.8–10.95% of which were methylated, was reliably about 10.5× to 13.8×, with differing levels of mCG, mCHG, and mCHH sites (Figure S3, Table S2).

To assess the influence of non-methylated cytosine on BS-seq library construction and control for methylcytosine sequencing preference [49], methylated loci mapped to unique sites in the reference genome were detected after M-bias assessment (Figure S4). The percentage of methylated cytosine to total cytosine, mC/C, was 13.72%, and the ratios of mCG/CG, mCHG/CHG and mCHH/CHH sites were 38.04%, 20.42% and 9.21%, respectively (Table 1). The relative proportion of mCG, mCHG, and mCHH sites throughout the genome was 28.28% (26.43–34.32%), 21.19% (20.24–24.46%), and 50.53% (41.42–53.26%), respectively (Figure 2b). Meanwhile, there existed a tendency toward mCG and mCHG sites with highly methylation levels; 70–100% were methylated with a large proportion. However, the mCHH context showed the opposite trend with a low methylation level; 0–30% were methylated with a large proportion (Figure S5). An examination of logo plots exploring methylation preferences at sites and nearby regions revealed no significant variations in mC preference (Figure S6). Chromosome 3 had the highest number of methylated sites of any chromosome (Figure S7).

**Figure 3:**
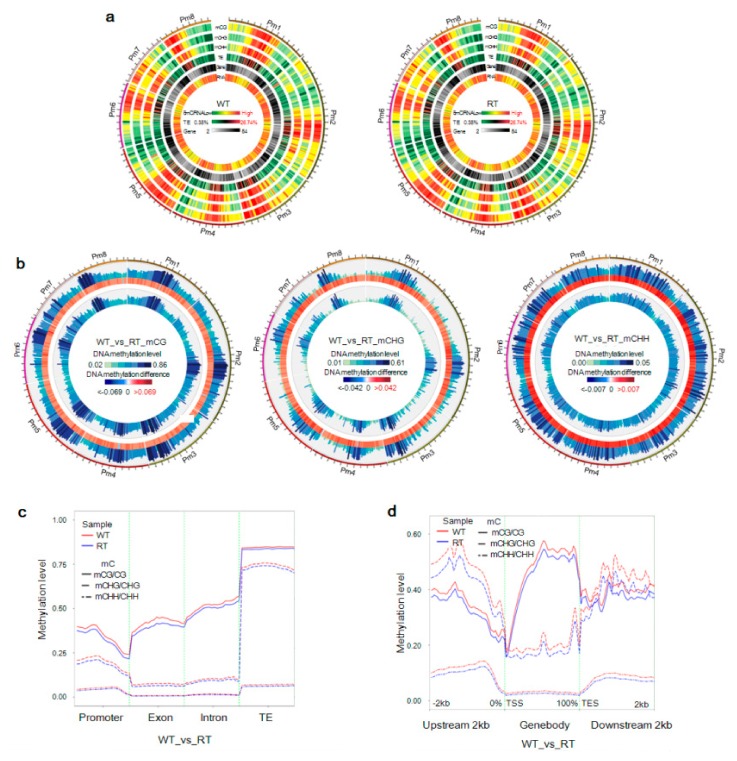
Epigenomic landscape and distribution of DNA methylation in white petal tissues (WT) and red petal tissues (RT) of *Prunus mume*. (**a**) Circos plots of chromosomes in *P. mume* WT and RT samples. The track order (from outside to inside) is as follows: (1–3) methylation density by sequence context (mCG, mCHG, and mCHH); (4) transposable element (TE) density; (5) gene density; and (6) number of reads generated by transcriptome sequencing. (**b**) Circos plots of differences in DNA methylation levels by methylation context (mCG, mCHG, and mCHH) in WT (outer track) and RT (inner track) genomes. The middle track in each plot indicates the degree of DNA methylation-level differences. (**c**) Distribution of DNA methylation levels within gene functional regions. The *x* and *y*-axes indicate gene functional domains and methylation levels, respectively. (**d**) Distribution of DNA methylation levels within gene-body domains and 2-kb upstream and downstream regions (TSS, transcriptional start site; TES, transcriptional end site). The *x* and *y*-axes indicate gene functional domains and methylation levels, respectively.

Methylation levels of CG, CHG, and CHH sites were highly correlated (*R*^2^ > 0.83) among different samples of the same petal color (Figure 2c), thus demonstrating that RT1–RT3 and WT1–WT3 could be analyzed as replicates of red petal tissue (RT) and WT samples, respectively. mCG, mCHG, and mCHH sites on chromosomes had similar distribution trends, with the most highly methylated regions in WT and RT corresponding to low-density genes (Figure 3a).

### 2.3. Levels of mC Variation in Different Colored Petal Tissues

The status and level of DNA methylation can vary between different individuals, tissues, and even genomic regions. We therefore explored variation in methylation between WT and RT samples in chromosomes as well as genomic regions. As shown in Figure 3b, the majority of strongly and weakly methylated regions occurred at similar chromosomal positions between the two samples. Calculated mCG/CG, mCHG/CHG, and mCHH/CHH ratios in the RT sample were 2.3–85%, 1.7–59%, and 0.4–4.3%, respectively. Many mC loci in RT were different from those in WT, where the mCG/CG, mCHG/CHG, and mCHH/CHH ratios were 2.8–83%, 1.7–61%, and 0.6–5%, respectively.

Although methylation levels in different genomic functional domains were higher in WT than in RT, they followed similar trends, with the highest and lowest methylation context ratios corresponding to mCG/CG and mCHH/CHH, respectively (Figure 3c). In the context of CG, methylation levels in exon and intron regions were higher than in promoter regions, but lower than in TE regions. For CHG and CHH, mCHG/CHG and mCHH/CHH ratios were lower in exon and intron regions than in promoter and transcriptional end site (TES) regions, although the difference in the mCHH/CHH ratio across various regions was not very pronounced. The highest methylation levels within gene bodies were at CG sites, whereas methylation levels in the ~2-kb upstream and downstream regions were highest at the CHG sites (Figure 3d). The lowest methylation levels, including at CG and CHG sites, were observed within regions adjacent to transcriptional start sites (TSSs) and TESs.

### 2.4. DMR-Related Genes and Bicolored Flowers on Individual Trees

**Figure 4:**
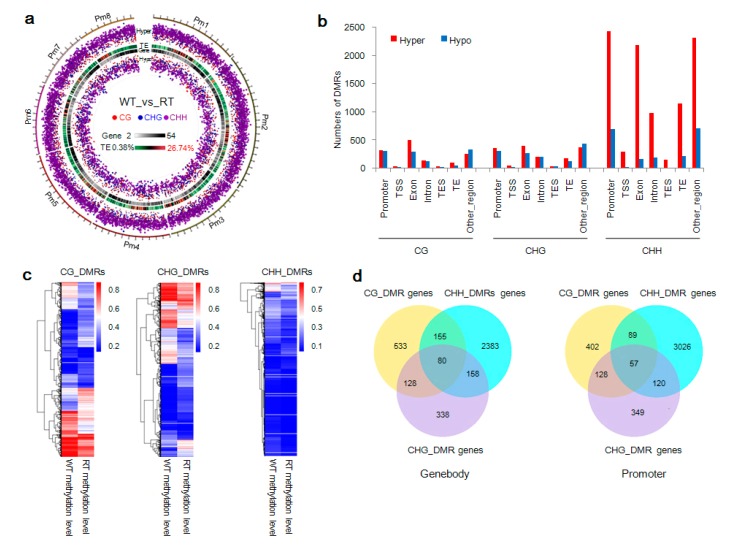
Distribution, methylation level, and predicted genes of differentially methylated regions (DMRs) in white petal tissues (WT) and red petal tissues (RT) samples. (**a**) Circos plots of CG, CHG, and CHH DMRs and transposable element (TE) and gene densities on each chromosome of *Prunus mume*. Track order (outside to inside) is as follows: scatter plot of hypermethylation (Hyper); TE density (TE); gene density (Gene); and scatter plot of hypermethylation (Hypo). Red, blue, and purple dots indicate CG, CHG, and CHH DMRs, respectively. (**b**) Distribution of CG, CHG, and CHH DMRs within gene functional regions. TSS, transcriptional start site; TES, transcriptional end site. (**c**) Heat maps of methylation levels of CG, CHG, and CHH DMRs. (**d**) Venn diagrams of predicted genes linked with CG, CHG, and CHH DMRs. “Genebody” and “promoter” indicate predicted genes anchored within gene body and promoter regions, respectively.

DMRs can be used to identify methylation differences between individuals or developmental stages and their involvement in gene transcriptional regulation [50,51]. In the present study, 13,468 DMRs—10,121 hypermethylated and 3347 hypomethylated—were predicted between WT and RT types using DSS software. These DMRs, whose lengths were normally distributed (Figure S8a), were methylated at levels of approximately 38.5% (CG), 31.5% (CHG), and 8.4% (CHH) (Figure S8b). DMR chromosomal distributions and levels of significance are displayed in Figure 4a and Figure S8c. In each gene functional region (except for other regions in CG and CHG contexts), the number of DMRs between WT and RT exhibiting hypermethylation was higher than those with hypomethylation. In the CHH context, many more hypermethylated DMRs were detected than hypomethylated ones, and the TSS and TES regions contained few DMRs (Figure 4b). Heat maps of methylation levels of CG, CHG, and CHH DMRs revealed that variation was present in the methylation of WT and RT samples (Figure 4c). As shown in Figure 4d, these DMRs were overlapped with 4376 gene bodies and 4622 gene promoters. A total of 80 genes containing mCG, mCHG, and mCHH sites in their transcribed region were detected; similarly, 57 gene promoters were predicted containing all three types of methylated sites.

Following DMR detection, Gene Ontology (GO) and Kyoto Encyclopedia of Genes and Genomes (KEGG) annotations were performed to explore the functions of DMR-related genes. These analyses uncovered the enrichment of 896 CG–DMR-anchored genes (543 hypermethylated and 361 hypomethylated), 704 CHG–DMR-anchored genes (432 hypermethylated and 288 hypomethylated), and 3777 CHH–DMR-anchored genes (2531 hypermethylated and 292 hypomethylated). In addition, 696, 654, and 3292 genes with promoters overlapping with CG, CHG, and CHH DMRs, respectively, were found to be enriched. The identified genes have important molecular functions in various biological processes, especially phenylalanine metabolism and the biosynthesis of phenylpropanoids, carotenoids, flavonoids, and plant hormones. Since these are critical processes in flower color formation, their over-representation among DMR-anchored genes suggests that variation in methylation levels of DMRs affected the color of WT and RT samples (Figures S9–S12).

### 2.5. Analysis of Differential Transcription between WT and RT

To reveal expression differences between WT and RT, we compared the transcriptomes of the six petal tissue samples. RNA-seq generated 44,202,732–55,656,090 clean reads per sample, of which 38,737,776–49,062,666 (88%; 85% unique) were mapped to the reference genome. Approximately half of the unique reads were mapped to the positive-sense strand of chromosomes (Figure S13, Table S3), and 294 transcription factors were detected (Table S4). Gene expression levels were distributed similarly, and significantly positively correlated among the sample genomes (*R*^2^ > 0.93) (Figure S14a,b). In the next step, the six samples were divided into two groups—WT (WT1, WT2, and WT3) and RT (RT1, RT2, and RT3)—for further analysis.

**Figure 5:**
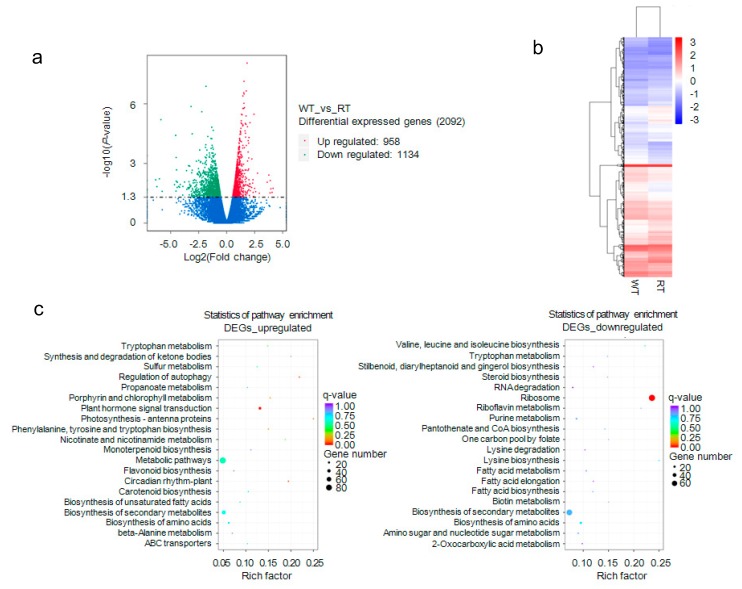
Analysis of differentially expressed genes (DEGs). (**a**) Detection and distribution of genes differentially expressed between white petal tissues (WT) and red petal tissues (RT). Green and red dots indicate upregulated and downregulated DEGs, respectively. (**b**) Cluster dendrogram of DEGs. (**c**) Kyoto Encyclopedia of Genes and Genomes (KEGG) pathway enrichment of upregulated and downregulated DEGs.

In this analysis, 16,383 expressed genes, 221 WT-specific and 765 RT-specific, were detected using a FPKM > 1 threshold (Figure S14c), with FPKM standing for fragments per kilobase of exon per million fragments mapped. Screening with DESeq yielded 958 upregulated and 1134 downregulated differentially expressed genes (DEGs) widely distributed across WT and RT genomes (Figure 5a,b). These DEGs belonged to 55 functional groups, including 36 biological process, 14 cellular components, and five molecular function categories. Many of the enriched genes take part in metabolic processes (Figure S14d). KEGG pathway analysis was used to further explore DEGs associated with anthocyanin biosynthesis and metabolic regulation (Figure 5c). We found that the genes were enriched mainly in pathways controlling plant hormone signal transduction (ko pmum04075), biosynthesis of secondary metabolites (ko pmum01110), metabolism (ko pmum01100), and flavonoid biosynthesis (ko pmum00941). Structural genes, including *Pm020453* (*YUCCA8*), *Pm004176* (*ANGLT*), *Pm031359* (*UGT79B6*), *Pm006139* (*GSTF1*), *Pm011195* (*GSTXC*), *Pm012985* (*GSTF7*), and *Pm025127* (*GSTX6*), were downregulated, but other critical genes, such as *Pm013782* (*DFRA*), *Pm018402* (*DFRA*), *Pm023202* (*DFRA*), *Pm017146* (*FLRT*), and *Pm008680* (*UGFGT*), were upregulated (Supplementary Data 1). In addition, 189 transcription factor genes, including *MYB*, *bHLH*, and *WD*, were detected using iTAK software (Supplementary Data 2), which suggests that transcription factors take part in the regulation of bicolored flowers.

### 2.6. Correlation between Gene Expression Levels and DNA Methylation

The DNA methylation of genes is correlated with changes in their expressions. To reveal the potential roles of expression level differences in WT versus RT regulated by DNA methylation, we analyzed the distribution of gene methylation and expression levels within each chromosome and gene functional region. As shown in Figure 3a, highly expressed genes were distributed in regions of low methylcytosine density. This negative correlation between gene expression levels and DNA methylation in general was apparent on scatter plots and heat maps (Figure S15).

**Figure 6:**
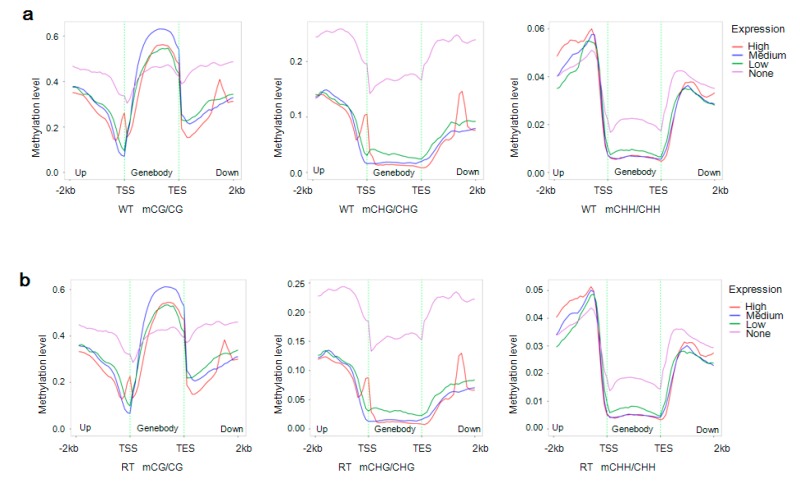
Distributions of methylation levels within gene bodies and their 2-kb upstream and downstream regions. Genes were classified into four groups according to their expression levels: no expression (pink; FPKM <1); low expression (green; 1≤ FPKM < FPKM_25%); medium expression (blue; FPKM_25% ≤ FPKM < FPKM_75%); and high expression (red; FPKM ≥ FPKM_75%). FPKM stands for fragments per kilobase of exon per million fragments mapped, and FPKM_25% and FPKM_75% refer to values at the boundary of the 25th and 75th percentiles of expression levels, respectively. Next, the different gene regions (gene body and 2-kb upstream and downstream) were divided into 50 bins, and the methylation levels of each were averaged. WT and RT indicate white petal tissues and red petal tissues, respectively. The *x* and *y*-axes represent gene body regions and DNA methylation levels by sequence context (mCG, mCHG, and mCHH), respectively. (**a**,**b**) Distributions of methylation levels within gene bodies and 2-kb upstream and downstream regions in samples WT [(**a**); FPKM_25% = 4.60, FPKM_75% = 45.31] and RT [(**b**); FPKM_25% = 4.91, FPKM_75% = 45.62].

To analyze the relationship between levels of expression and methylation within gene bodies (including 2-kb upstream and downstream domains), we divided genes into high, medium, low, and no expression groups [high expression (FPKM ≥ FPKM_75%); medium expression (FPKM_25% ≤ FPKM < FPKM_75%); low expression (1 ≤ FPKM < FPKM_25%); and no expression (FPKM < 1). FPKM stands for fragments per kilobase of exon per million fragments mapped, and FPKM_25% and FPKM_75% refer to values at the boundary of the 25th and 75th percentiles of expression levels, respectively]. And their methylation levels were plotted as a function of location [48]. As shown in Figure 6, the non-expressed genes displayed high methylation levels at CG sites within domains that are 2-kb upstream or downstream of the gene body and at CHG sites in all of the gene regions. Within the gene body and 2-kb downstream regions, non-expressed genes showed high methylation levels at CHH sites, but gene expression was positively correlated with CG methylation within the gene body. Similarly, a positive correlation was detected between gene expression and CHH methylation levels within 2-kb upstream of the TSS. Interestingly, two peaks in CG and CHG methylation levels were observed in the 2-kb upstream and downstream regions of highly expressed genes.

**Figure 7:**
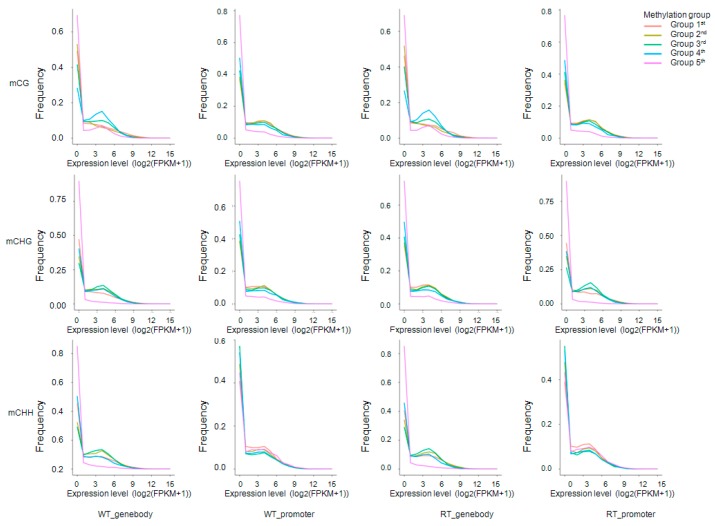
Frequencies of expressed genes anchored by methylation sites within gene bodies and promoters. For each methylation sequence context (mCG, mCHG and mCHH), expressed genes were divided into five groups according to methylation level: group 1 (red; methylation level < level_20%); group 2 (yellow-green; level_20% ≤ methylation level < level_40%); group 3 (green; level_40% ≤ methy_level < level_60%); group 4 (blue; level_60% ≤ methy_level < level_80%); group 5 (pink; methy_level ≥ level_80%). Level_20%, _40%, _60%, and _80% represent values at the boundaries of the 20th, 40th, 60th, and 80th percentiles of methylation levels. WT and RT indicate white petal tissues and red petal tissues, respectively. The *x* and *y*-axes represent gene expression levels and gene frequencies, respectively.

Another statistical approach, projecting methylation levels onto a coordinate system of expression levels (*x*-axis) and frequency (*y*-axis), was used to further explore gene expression and DNA methylation (Figure 7). We divided the methylation levels of gene bodies and promoters into five groups according to Xu et al. [48], and found that DNA methylation and gene expression levels were negatively correlated in most genes. In contrast, genes whose promoters lacked mCHH sequences were only weakly expressed (Figure 7); this situation may have been responsible for the positive correlation between gene expression and methylation levels within 2-kb upstream regions (Figure 6).

### 2.7. DEGs with Methylation Modification and DMR-Related Gene Expression

**Figure 8:**
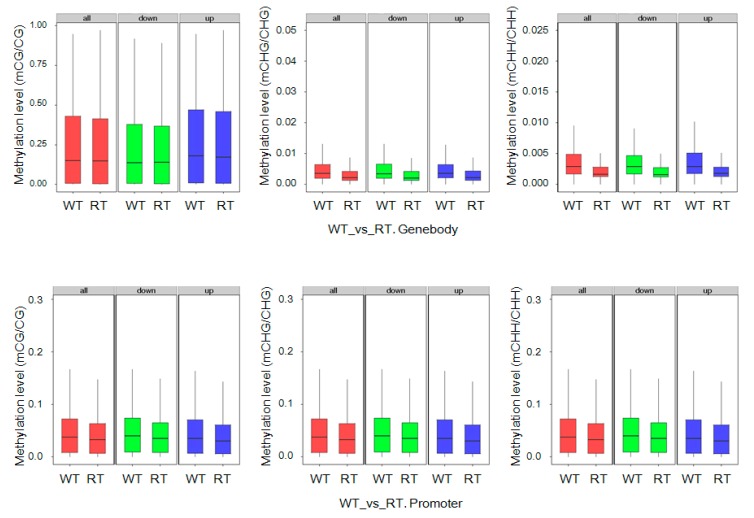
Methylation levels of differentially expressed genes (DEGs) by sequence context (mCG, mCHG, and mCHH) and gene region in white petal tissues (WT) and red petal tissues (RT). The terms “all”, “up”, and “down” indicate all, upregulated, and downregulated DEGs, respectively. DNA methylation levels within gene bodies and promoters of genes differentially expressed between WT and RT are shown (“WT vs. RT. Genebody” and “WT vs. RT. Promoter”, respectively).

Analysis of DEG DNA methylation levels revealed large differences between WT and RT (Figure 8). In WT samples, high methylation levels were observed within DEGs, including downregulated and upregulated ones (i.e., involving promoters), at CG, CHG, and CHH sites. In contrast, DEGs in RT samples exhibited low methylation levels, with especially low levels observed at CHG and CHH sites within gene body domains. We also observed that DEG expression level differences between WT and RT samples were positively correlated with ratios of mCG/CG (*r* = 0.037, *p*< 0.01), mCHG/CHG (*r* = 0.046, *p*< 0.01), and mCHH/CHH (*r* = 0.037, *p*< 0.01) in gene body regions. However, in 2-kb downstream regions, a negative correlation (*r* = −0.038, *p*< 0.01) was observed between CHG methylation and expression level differences between WT and RT (Figure S16).

**Figure 9:**
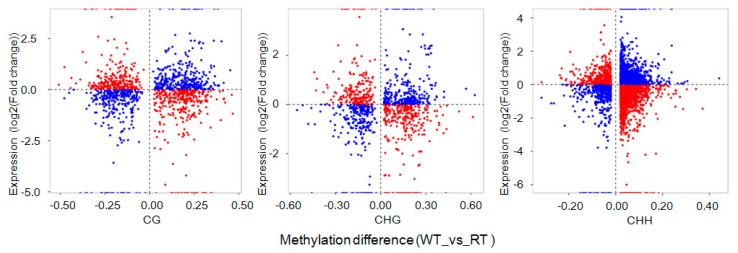
Distributions of differential expression of differentially methylated region (DMR)-related genes between white petal tissues (WT) and red petal tissues (RT). CG, CHG, and CHH refer to CG-DMR, CHG-DMR, and CHH-DMR-related genes, respectively. The *x*-axis indicates methylation-level differences, and the *y*-axis indicates gene differential expression levels. Red dots represent hypermethylated (hypomethylated) DMR-related genes associated with downregulated (upregulated) expression. Blue dots represent hypomethylated (hypermethylated) DMR-related genes associated with upregulated (downregulated) expression.

**Figure 10:**
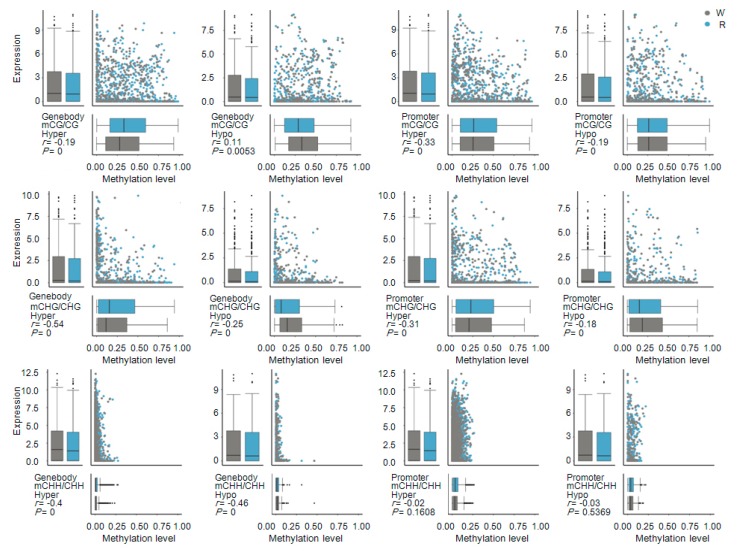
Combined maps of correlations between differentially methylated region (DMR)-related genes and expression levels. Each of the 12 subfigures is divided into four sections: upper left, box plot of DMR-related gene expression; upper right, scatter plot of DMR-related gene expression vs. methylation level; bottom left, comparison and correlation statistics; bottom right, box plot of DMR-related gene methylation level. WT and RT indicate white petal tissues (gray) and red petal tissues (blue), respectively.

Next, an expression analysis of DMR-related genes (located in both gene body and promoter regions) identified 1154 mCG–DMR-related genes, 852 mCHG–DMR-related genes, and 4282 mCHH–DMR-related genes. Hypomethylated DMR-related genes were mostly associated with highly expressed genes, whereas the hypermethylation of DMR-related genes was associated with decreased expression levels (Figure 9). Methylation levels of DMR-related genes were negatively correlated with their expression levels with one exception: hypomethylation at CG sites within gene body regions was positively correlated with gene expression (Figure 10).

### 2.8. Gene Ontology (GO) and Kyoto Encyclopedia of Genes and Genomes (KEGG) Enrichment of DMR-Related Genes Associated with DEGs

**Figure 11:**
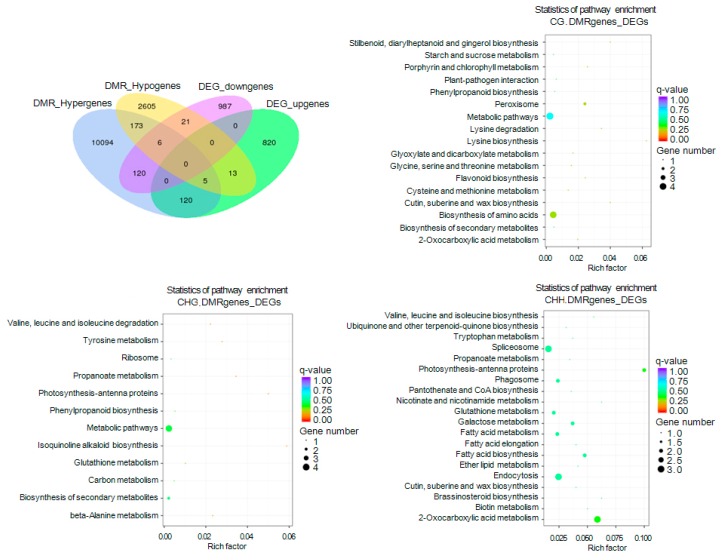
Venn diagram and KEGG pathway enrichment of differentially methylated region (DMR)-related differentially expressed genes (DEGs). DEGs are anchored by DMRs within gene body regions. DMR_Hypergenes and DMR_Hypogenes indicate hypermethylated and hypomethylated DMR-related genes, respectively. DEG_downgenes and DEG_upgenes indicate downregulated and upregulated DEGs, respectively. CG.DMRgenes, CHG.DMRgenes, and CHH.DMRgenes refer to CG-DMR, CHG-DMR, and CHH-DMR-related genes, respectively.

To investigate the effect of DNA methylation on genes with unique functions, we performed GO and KEGG enrichment analyses of gene sets generated from an association analysis between DMR-related genes and 506 DEGs. We first identified 285 DMR-related genes associated with gene-body domains of downregulated or upregulated DEGs. Of these 285, 126, and 125 hypermethylated DMR-related genes were associated with upregulated and downregulated DEGs, respectively, whereas 27 and 18 hypomethylated DMR-related genes were associated with upregulated and downregulated DEGs, respectively. In addition, six upregulated and five downregulated DEGs overlapped with both hypermethylated and hypomethylated DMR-related genes (Figure 11). We also discovered that 282 associated DEGs, 61 of which also overlapped with DMR-related genes within gene body regions, were hypermethylated (213) or hypomethylated (83) within promoter domains. A total of 43 and 40 DMR-related genes that were hypomethylated within promoter regions were associated with downregulated and upregulated DEGs, respectively; these numbers were larger than the number of genes hypomethylated within promoter domains (Figure S17).

GO enrichment analysis was then carried out to functionally classify the DEGs associated with DMR-related genes into biological process, cellular component, and molecular function categories (Figure S18). We also performed a KEGG analysis on the associated DEGs, which revealed the enrichment of the following pathways that play important roles in flower color formation: flavonoid biosynthesis (ko pmum00941), the biosynthesis of secondary metabolites (ko pmum01110), phenylpropanoid biosynthesis (ko pmum00940), and plant hormone signal transduction (ko pmum04075) (Figure 11, Figures S17, S19 and S20). Examination of methylation level modifications of key structural and transcription factor genes differentially expressed between WT and RT indicated that *Pm031359* (*UGT79B6*) was hypermethylated at CHH sites in exon regions, and *Pm027422* (*YUCCA2*) and *Pm008425* (*MYB305*) were hypermethylated at CHH sites in the promoters of downregulated genes. In upregulated DEGs, *Pm008680* (*UFGT*) and *Pm019063* (*CHI*) were hypomethylated at CG and CHH sites within exons and CG sites within promoters. Hypermethylation was also detected in promoter and intron regions of the *Pm013782* (*DFRA*) gene (Supplementary Data 1).

### 2.9. Detection of Methylated TEs Regulating Candidate Gene Expression

**Figure 12:**
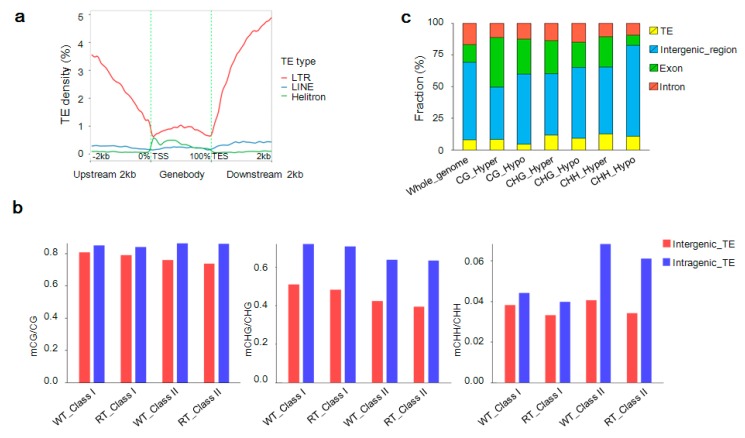
Methylation of transposable elements (TEs) in the genomes of *Prunus mume*. (**a**) Distribution of TEs density within gene body and 2-kb upstream and downstream domains. (**b**) Distribution of methylated TEs according to sequence context (mCG, mCHG, and mCHH) in intergenic (red) and intragenic (blue) domains. WT and RT indicate white petal tissues and red petal tissues, respectively. Class I and class II refer to class-I and class-II TEs, respectively. (**c**) Genome-wide length percentages of differentially methylated regions (DMRs) within TEs and other different functional regions (leftmost bar) and the methylome-wide percentage distributions of DMRs with different methylation types in different functional regions.

At the global genome level, TE density was heaviest in genomic regions with a high methylation density and lightest in areas of low gene density and low gene expression (Figure 3a). Most long terminal repeat (LTR) TEs were located in 2-kb upstream and downstream regions of gene body domains, while *Helitron* TEs were mainly scattered throughout the gene body and long interspersed nuclear elements (LINE) were approximately evenly distributed (Figure 12a). Class-II TEs in intragenic regions were the most heavily methylated of the intergenic and intragenic class-I and class-II TEs. In addition, more methylated sites were found within TEs in the WT sample than in the RT sample. An analysis of the methylation sequence context of TEs revealed that mCG and mCHG accounted for a large proportion of methylated sites, especially within intragenic areas (Figure 12b).

A total of 1833 DMRs (1437 hypermethylated and 396 hypomethylated) comprising 158 mCG, 302 mCHG, and 1373 mCHH sequences were detected within TEs between WT and RT samples. Hypermethylated mCG, hypomethylated mCG, hypermethylated mCHG, hypomethylated mCHG, hypermethylated mCHH, and hypomethylated mCHH within TEs accounted for 8.3%, 4.8%, 11.6%, 9.2%, 12.6% and 10.7%, respectively, of DMRs in genomic functional regions (Figure 12c). Among these DMRs, 197 were located in 196 TEs inserted into 106 DEGs associated with DMR-related genes (Supplementary Data 3). The gene *Pm008680* (*UGFGT*), with three hypomethylated-mCHH *Copia* TE insertions, was upregulated in WT relative to RT, as was *Pm013782* (*DFRA*) carrying three inserted hypermethylated TEs (two hypermethylated-mCHG *L1* and one hypermethylated-mCHG *Copia* elements). In contrast, the *Pm031359* (*UGT79B6*) gene, harboring a hypermethylated-mCHH *Copia* TE insertion in its 2-kb upstream region, and the *Pm031359* (*UGT79B6*) gene, with a hypermethylated-mCHG *Helitron* TE insertion in its 2-kb downstream region, were both downregulated (Supplementary Data 1).

## 3. Discussion

Since variation in petal color is highly prized in ornamental plant species, *P. mume* trees bearing chimeric red and white flowers have been selected during the process of genetic improvement. Floral chimerism is caused by the spatially and temporally restricted deposition of plant pigments, for instance, secondary metabolites of anthocyanins [7,25,37,52,53]. In our study, we detected Cy3G, Cy35G, and Pn3G in RT samples by HPLC-MS. The implied function of these compounds as determinants of color in red petal tissues suggests that genes related to the anthocyanin regulatory pathway, as revealed by an association analysis between transcriptomes and methylomes, are the molecular basis of chimerism in *P. mume* “Danban Tiaozhi”.

### 3.1. Genes within Anthocyanin Regulation Pathway Were Differentially Expressed

Numerous molecular studies have revealed the processes controlling the genetics of plant organ coloration, which include differences in expressions of structural and transcription factor genes. For example, Han et al. [54] have reported that the downregulation of *CHI* and *DFR* by *anthocyanidin reductase* (*ANR*) results in yellow-skinned apple fruits. In bicolored flowers of *Petunia hybrida*, the enhancement of *CHS* expression induces blue or red coloration, respectively, in blue–white or red–white sectors of variegated flowers [5,23]. RNA-Seq technology has revealed that the significant upregulation of annotated anthocyanin biosynthetic genes *CHS*, *F3H*, *F3H*, *DFR*, *leucoanthocyanidin dioxygenase* (*LDOX*), *ANS*, and/or *UF3GT* is responsible for purple flesh coloration in a *Dioscoreaalata* cultivar, bicolored tepals in lily, and variegated petals in *P. mume* “Fuban Tiaozhi” [7,20,53]. In our study, we found that the anthocyanin biosynthetic gene homologs *Pm020453* (*YUCCA8*), *Pm004176* (*ANGLT*), and *Pm031359* (*UGT79B6*) were upregulated in red petal tissues, but other critical genes, such as *Pm013782* (*DFRA*), *Pm018402* (*DFRA*), *Pm023202* (*DFRA*), *Pm017146* (*FLRT*), and *Pm008680* (*UGFGT*) were downregulated. These differences between *P. mume* “Danban Tiaozhi” and “Fuban Tiaozhi” may be due to the temporal and tissue-specific nature of transcriptome expression [7], which has different development stages, and its genotypes show a variety of gene expression [8]. We also discovered that *GST* genes, which encode proteins transporting cyanidins and/or anthocyanins to the tonoplast [55], were upregulated in red tissues. This result is similar to the finding of a previous study that the *Riant* gene encoding a GST protein induces red flower coloration in peach [24].

The identification and functional characterization of flavonoid-related R2R3-MYB transcription factors, which show active or repressive effects on anthocyanin biosynthetic genes, is important for revealing plant pigmentation [56]. A study of blood-fleshed *P. persica* uncovered a mechanism whereby *BLOOD* (*BL*) was the key gene for the blood-fleshed trait via its activation of *PpMYB10.1*, and the silencing of *BL* reduced anthocyanin pigmentation in maturing fruits [6]. The MYB10 promoter is more variable in *Malus* × *domestica* “Honeycrisp” than in “Royal Gala”, which results in a more variable color pattern in the peel of the first cultivar [36]. A transcriptomic comparison of red and green-colored leaves of *P. persica* has identified a MYB transcription regulator, *PpMYB10.4*, whose transient expression induces anthocyanin accumulation [57].

In “Lollypop” Asiatic lilies, the transcriptional profiling of *LhMYB12* has revealed that the presence of bicolored tepals is controlled by the transcriptional regulation of anthocyanin biosynthetic genes [53]. Differential expression of *Peace* (*peach anthocyanin colour enhancement*, a *R2R3 MYB-like* gene) determines the pattern of flower coloration in variegated petals within individual trees of flowering *P. persica* “Genpei” [25]. In addition, jasmonate has been reported to regulate WD-repeat/bHLH/MYB complex-mediated anthocyanin accumulation in *Arabidopsis thaliana* [58]. Using iTAK software, we detected 189 differentially expressed transcription factor genes, including *MYB*, *bHLH*, and *WD*, suggesting that transcription factors may play important roles in the formation of chimeras in *P. mume* “Danban Tiaozhi”.

### 3.2. Methylcytosine Modification Affected the Expression of Anthocyanin-Related Genes

Cytosine methylation is a common form of DNA modification that is closely interwoven with the process of gene transcription. Although important for the epigenetic regulation of endogenous genes, the extent to which this type of DNA modification regulates genomes remains elusive [59,60]. DNA methylation occurs at higher levels in heterochromatin than euchromatin, and performs specific functions across different species [43,48,61,62]. In this study, we mapped the first-reported methylomes of *P. mume* flower petals using the single base resolution technique. We also compared methylomes and transcriptomes to elucidate the relationship of methylcytosine and gene expression. We discovered that 11.29–14.83% of cytosine sites within genomes were methylated, and the patterns of methylation (mCG, mCHG and mCHH) were similar to those reported in *Arabidopsis* [60,63], rice [64,65], maize [66], soybean [44,67], tomato [43], apple [48], and cotton [45,47]; specifically, the highest and lowest levels of methylation were at the CG and CHH sites, respectively, with significant differences observed within both WT and RT tissues. We also found that the mCG/CG ratios were highest in exon and intron regions; in contrast, mCHG/CHG and mCHH/CHH ratios were lower in the exon and intron regions than in the promoter and TE regions.

Gene transcription is influenced or regulated by DNA methylation [59,68], with methylcytosine levels negatively correlated with gene expression in general [69]. In our study, we observed a similar pattern. We also found that methylcytosine levels of RT samples were lower than those of WT samples, which suggests that anthocyanidin-related genes are suppressed by the high levels of methylcytosine in WT genomes. However, a contradictory result was seen in gene body regions, where methylcytosine levels of hypomethylated CG-DMR-related genes were positively correlated with gene expression. This latter observation is consistent with the finding that genes methylated in transcribed regions are highly expressed and constitutively active in *A. thaliana* [60], and that CG methylation is often linked to increased gene expression [68,70,71].

Next, we searched for DMRs and investigated the expression of DMR-related genes (especially DEGs). We focused on DMRs in functional regions, as gene body methylation is conserved across species among constitutively expressed genes [60,64,71], and because methylation within promoters is highly tissue-specific in nature and strongly associated with transcriptional repression in plants [60,69,72]. We detected 13,468 DMRs associated with 4376 gene bodies (285 DEGs) and 4622 gene promoters (282 DEGs). These associated DEGs were enriched in KEGG terms such as flavonoid biosynthesis, the biosynthesis of secondary metabolites, phenylpropanoid biosynthesis, plant hormone signal transduction, and transcription factor activity. In previous studies, gene methylation has been observed to influence floral pigmentation. For instance, red pigmentation is observed in the flowers of the transgenic petunia line 17-R upon hypomethylation of the 35S promoter driving the *A1* gene [26], while mosaic red anthocyanin in lip crests, sepals, and petals of yellow flowers of *Oncidium* “Gower Ramsey” may be attributed to activation of the *OgCHS* gene resulting from the demethylation of the five-upstream promoter region [27]. As a third example, methylation levels of *MYB* genes are associated with the formation of red-skinned pears and apples [37,38]. Thus, we suggest that the methylation or demethylation of genes participating in the anthocyanin regulation pathway is responsible for flower color chimerism in *P. mume* “Danban Tiaozhi”.

### 3.3. TEs with Methylcytosine Affected the Expression of Anthocyanin-Related Genes

Various evidence supports the role of TE insertions in gene expression changes and phenotypic variation in higher plants. In anthocyanin biosynthetic genes, for example, TE-induced insertions cause null mutations that result in variations in seed, peel, and flower coloration. The insertion and excision of the *Ds* transposon has given rise to variation in the size and intensity of colored spots in maize kernels, and a *Candystripe 1* insertion in the second intron domain of the *y-candystripe* allele has altered the pigmentation of the sorghum grain pericarp from solid red to variegated [8,73,74]. Similarly, the *TRANSPARENT TESTA8* (*BrTT8*) locus, encoding a bHLH protein, lost its function with the insertion of a *Helitron* transposon, resulting in yellow seeds in *Brassicarapa* [75]. The golden pigmentation in hulls and internodes of *Oryza sativa* mutants is due to the complete suppression of the *OsCHI* gene following insertion of a *Dasheng* retrotransposon into its 5′ untranslated region (UTR), while a retrotransposon insertion in the upstream sequence of the pigmentation-related gene *VvmybA1* is regarded as the molecular basis for white-skinned coloration in grape cultivars [15,76]. TE-mediated insertional mutations have also caused alterations in seed coat and flower color in both *Ipomoea purpurea* and *Glycine max* [77,78,79,80]. Transformed tobacco plants carrying an inserted *Tag1* element between the *CaMV* 35S promoter and the maize *R* gene have variegated flowers, and the insertion of either *Ty1dic1* or *Retdic1* transposons can disrupt the *AA5GT* (*acyl-glucose-dependent anthocyanin 5-O-glucosyltransferase*) gene to prevent glycosylation of the 5′ position of anthocyanins in *Dianthus caryophyllus* [14,17].

One unanswered question concerns how TEs are activated or repressed to ensure a stable phenotype. Emerging evidence is demonstrating that TEs are silenced or reactivated by epigenetic mechanisms such as DNA methylation modification [39,41,42,68,81,82]. In the model plant *A. thaliana*, the imprinted gene *FWA* is a flowering-time modifier, with its silencing dependent on the cytosine methylation of a SINE retro element in the promoter region [83]. Transposons in *Arabidopsis* are heavily methylated at both CG and non-CG sites, whereas non-CG methylation is rarely found in active genes [70]. In rice, the essential *chloroplast protease 5* (*OsClpP5*) gene with the insertion of an epigenetically silenced *autonomous DNA-based active rice transposon 1* (*aDart1*) may induce leaves to show variegation [84]. Similarly, in our study, we detected 197 DMRs associated with 196 TEs inserted into 106 DEGs. We annotated these DEGs with GO and KEGG functional terms and pathways. The genes *Pm008680* (*UGFGT*), *Pm031359* (*UGT79B6*), *Pm031359* (*UGT79B6*), *Pm013782* (*DFRA*), and *Pm011195* (*GSTXC*), all participating directly in the color regulation pathway and anthocyanin transport, were enriched. This result suggests that the insertion of methylcytosine-modified transposons affects the expression of anthocyanin genes, resulting in chimerism.

## 4. Materials and Methods

### 4.1. Plant Materials

White and red petal tissues were collected from fresh flower blossoms of the flower color chimera *P. mume* “Danban Tiaozhi” on a fine day between 9:00–11:00. The collected tissues were carefully sorted under subzero temperatures with fine-tipped tweezers into six groups according to their color and origin (Figure 1a). Tissues in the first two groups came from individual flowers having both white (WT1) and red (RT1) petal tissues. The second two groups individually consisted of white (WT2) and red (RT2) petal tissues from single-color (white or red) flowers that were located together in the same branches. The final two groups comprised petal tissues derived from separate branches bearing either white (WT3) or red (RT3) flowers only. To facilitate the use of these materials for biochemical detection and a variation analysis of single base resolution genomes, all of the tissues were harvested from a single ornamental tree, snap-frozen in liquid nitrogen, and stored at −80 °C.

### 4.2. Qualitative and Quantitative Analysis of Floral Pigments

Previously frozen petal tissues were ground into fine powder in liquid nitrogen. Next, 200.0-mg portions of individual petal tissue samples were placed in centrifuge tubes containing extraction reagent (70:27:2:1 (*v*/*v*/*v*/*v*) methanol-water-formic acid-trifluoroacetic acid) [85] and vortexed for 1 min at room temperature followed by ultrasonic extraction in a KQ 2200B ultrasonic cleaner (Jiangsu, China) at 4 °C for 15 min. After storage overnight at −20 °C, the mixtures were centrifuged (A-14C, Sartorius, Goettingen, Germany) for 10 min at 12,000 rpm and 4 °C. Supernatants were collected and filtered through 0.22-μm nylon membranes [86] for high-performance liquid chromatography-electrospray ionization-mass spectrometry (HPLC-ESI-MS) analysis.

Preliminary HPLC analysis [86] was performed on a Waters 2695 system (USA) equipped with a W2996 photodiode array and a C18 column (5 μm, 4.6 × 250 mm i.d.; WondaCract ODS-2, Shimadzu, Shanghai, China). The major parameters were set as follows: column temperature = 30 °C, absorption spectrum = 200 to 600 nm, injection volume = 10 μL, and flow rate = 0.8 mL/min. Gradient separation was carried out using a two-solvent system, 0.5% formic acid in water (phase A) and 0.1% formic acid in acetonitrile (phase B), as follows: 0 min, 5% B; 5 min, 10% B; 20 min, 20% B; 30 min, 25% B; 33 min, 10% B; 35 min, 5% B; and 50 min, 5% B. Quantification of WT and RT samples was performed using three replicates (WT1, WT2 and WT3, and RT1, RT2 and RT3, respectively) and an external standard. The external standard was cyanidin 3-*O*-glucoside chloride (Sigma-Aldrich, St. Louis, MO, USA), which was dissolved in the same extraction reagent (70:27:2:1 (*v*/*v*/*v*/*v*) methanol-water-formic acid-trifluoroacetic acid) with a concentration of 0.125 mg/mL.

Following preliminary HPLC identification, anthocyanins were detected using an HPLC system (Agilent 1200LC, Santa Clara, CA, USA) equipped with a diode array detector at 520 nm and the same C18 column used above and coupled to an electrospray ionization-mass spectrometer (ESI-MS) (6310 MSD Trap VL, Agilent, USA). ESI-MS was performed with the following settings: positive ionization mode (ESI, *m*/*z* 50–1000 mass units), gas temperature = 350 °C, flow rate = 8.0 L/min, nebulizer pressure = 35 psi, and capillary exit voltage = 120.4 V.

### 4.3. Bisulfite Sequencing and DMR Analysis

#### 4.3.1. Extraction of DNA and BS-Seq

WT1, WT2, WT3, RT1, RT2, and RT3 samples were separately ground into fine powder in liquid nitrogen. Genomic DNA was extracted using a DNeasy Plant Mini kit (QianGen, Shanghai, China) following the manufacturer’s instructions and then checked on 0.1% agarose gels and a NanoPhotometer spectrophotometer (Implen, Westlake Village, CA, USA). DNA concentrations were determined with a Qubit DNA Assay kit on a Qubit 2.0 fluorometer (Life Technologies, Carlsbad, CA, USA).

Next, 5.2 μg of qualified DNA spiked with 26 ng of lambda DNA (negative control) was sheared in a Covaris S220 ultrasonicator into random 200–300-bp fragments. The resulting fragments were then subjected to end repair, adenylation, and methyl-treated adapter ligation. Two bisulfite treatments with an EZ DNA Methylation-Gold kit (Zymo Research, Irvine, CA, USA) were applied to these fragments to transform non-methylated cytosines into uracil for subsequent base pairing with thymine by PCR. After PCR amplification using KAPA HiFi HotStart Uracil + ReadyMix (2×), the generated BS-seq library was quantified on a Qubit 2.0 fluorometer (Life Technologies) and by quantitative PCR, and insert size was assayed on an Agilent Bioanalyzer 2100 system. Sequencing of the BS-seq library, which generated 125/150-bp paired-end reads, was performed on an Illumina Hiseq 2500 platform followed by Illumina CASAVA pipeline analysis.

#### 4.3.2. Quality Assessment of Sequencing Data

The sequenced paired-end reads (raw reads or raw data) were checked for quality using FastQC (fastqc_v0.11.5) and stored in the FASTQ file format (Babraham Bioinformatics, http://www.bioinformatics.babraham.ac.uk/projects/fastqc/). The resulting files were pre-processed with Trimmomatic v0.36 software [87] using the following parameters: SLIDINGWINDOW: 4:15; LEADING:3, TRAILING:3; ILLUMINACLIP: adapter.fa: 2:30:10; and MINLEN:36. Reads passing these filtering steps were counted as clean reads for use in subsequent analyses. Finally, basic quality statistics on the clean reads were obtained using FastQC.

All the sequencing data (accession number: CRA000731) are available in the database of Genome Sequence Archive (GSA, http://bigd.big.ac.cn/).

#### 4.3.3. Reference Genome Preparation and Mapping of Clean Reads

To enable the mapping of clean reads, we first prepared a reference genome of *P. mume* [1]. Then, we performed reverse complementation process (C to T, and G to A) using Bismarkv 0.16.3 software [88] and Bowtie2 [89]. In addition, a gene annotation file in gene transfer format, a Gene Ontology (GO) annotation file, a description file, and a gene region file in browser extensible data format were generated for subsequent annotation and function analyses.

The quality-checked clean reads generated by BS-seq were aligned against the two strands of the converted reference genome (X700-dovetail). The best unique alignment of these sequence reads was selected from the two sets of pairwise comparisons. To infer cytosine methylation states and positions, the sequences were then compared against the normal genomic sequence. Identical sequences aligned to a unique genomic region were regarded as duplicates and used to estimate sequencing depth and coverage. To allow their visualization in the IGV browser, sequences were transformed into bigWig format (non-overlap) [88,90]. The bisulfite non-conversion rate was defined as the number of sequenced cytosines at all of the cytosine reference positions divided by the number in the lambda genome.

#### 4.3.4. Evaluation of Methylation Level and Distribution

Methylation level was calculated as ML(mC) = reads(mC)/(reads(mC) + reads(C)), where mC is methylcytosine, C is non-methylated cytosine, and ML(mC) is the methylcytosine level. As recommended in a previous study [91], the parameter ML(mC) was corrected to ML(corrected) according to the following formula: ML(corrected) = (ML(mC) − r)/(1 − *r*), where *r* represents the bisulfite non-conversion rate. Methylcytosine sequence contexts—mCG, mCHG, and mCHH (where H represents A, T, or C)—were analyzed. Methylation level densities and methylcytosine distributions in each chromosome and gene functional region (promoter, exon, intron, and 2-kb upstream and downstream regions) were also analyzed [43,92,93]. Differences in global methylation levels and methylcytosine distributions in gene structural regions (including 2-kb upstream and downstream) were compared between samples [72]. To focus on petal color variation, WT1, WT2, and WT3 samples were merged together as three biological replicates of WT; similarly, RT1, RT2, and RT3 served as the three RT biological replicates.

#### 4.3.5. Correlation Analysis and DMR Detection

A correlation analysis was carried out based on Pearson’s coefficient [94]. DSS software was used to identify DMRs and differentially methylated loci between WT and RT samples [95,96,97]. Information from neighboring cytosine sites (i.e., spatial correlation) and site read depths were analyzed to improve the accuracy of long cytosine reads. Variance among biological replicates was analyzed using a beta-binomial distribution model. DMRs were annotated, and DMR-related genes were defined as those having coding regions (from the transcriptional start site (TSS) to the transcriptional end site (TES)) or promoter regions (i.e., upstream 2-kb from the TSS) that overlapped with the distribution of DMRs.

#### 4.3.6. GO and KEGG Enrichment Analysis of DMR-Related Genes

GO enrichment analysis of DMR-related genes was performed using the GOseq R package [98], which also corrects for gene length bias. A GO term was considered to be significantly enriched in DMR-related genes at a corrected *p*-value threshold of 0.05. KEGG analysis (http://www.genome.jp/kegg/), an approach for understanding high-level functions and relationships in biological systems, was applied to uncover the pathway enrichment of DMR-associated genes [99]. KOBAS software [100] was used to test for statistical enrichment of DMR-related genes, which were then subdivided into all, hypermethylation, and hypomethylation categories and assigned to KEGG pathways.

### 4.4. Transcriptome Sequencing and Differentially Expressed Gene (DEG) Analysis

#### 4.4.1. RNA Isolation and Sequencing

RNA was isolated from WT1, WT2, WT3, RT1, RT2, and RT3 samples using an RNeasy Plant Mini kit (QianGen). Extracted RNA was checked for degradation and contamination using 1% agarose gels and a NanoPhotometer spectrophotometer (Implen, CA, USA), respectively, and then quantified with a Qubit RNA Assay kit on a Qubit 2.0 fluorometer (Life Technologies). RNA integrity was assessed on a Bioanalyzer 2100 system (Agilent) using the supplied RNA Nano 6000 assay kit.

For sequencing library construction, first and second-strand cDNA synthesis was carried out using 3 µg of RNA and a NEBNext Ultra RNA Library Prep Kit for Illumina (NEB, Ipswich, MA, USA), following the manufacturer’s instructions. After purification using an AMPure XP system (Beckman Coulter, Beverly, MA, USA), the synthesized strands were subjected to three-end adenylation and the addition of a poly-A tail and a NEBNext adapter. Next, 150–200-bp adapter-ligated fragments were preferentially selected using AMPure XP beads and amplified by PCR. The quality of the enriched cDNA library was assessed on an Agilent Bioanalyzer 2100 system. Clusters were generated from the qualified library using a cBot Cluster Generation System with a TruSeq PE Cluster kit v3-cBot-HS (Illumina, San Diego, CA, USA) and then sequenced on an Illumina Hiseq platform to generate 125-bp/150-bp paired-end reads (raw reads).

#### 4.4.2. Mapping and DEG Analysis

After quality control to remove low-quality reads and adapter contaminants from the raw reads, the remaining clean reads were aligned to the *P. mume* reference genome [1] using HISAT2.0.4 software with default parameters [101]. Cufflinks v2.1.1 was then used to assemble and identify known and novel transcripts, and HTSeq v0.6.1 (-m union) was used to estimate gene expression levels based on fragments per kilobase of transcript sequence per millions of base pairs (FPKM) [102]. Differential expression analysis of WT and RT groups, with three biological replicates per group, was performed in DESeq v1.10.1, a program providing statistical routines for the determination of differential expression in digital gene expression data with a negative binomial distribution (Kij~NB(μij,σij2)) [103,104]. Significant DEGs were identified using an adjusted *p*-value cutoff of 0.05 and then subjected to GO [98] and KEGG [100] annotation.

### 4.5. Identification of Transcription Factors and TEs

Transcription factors and transcriptional regulatory factors were predicted and classified from the assembled transcriptome data according to Pérez-Rodríguez et al. [105] and Jin et al. [106]. In addition, the DEG sequences were screened for transcription factors using the iTAK tool (http://bioinfo.bti.cornell.edu/tool/itak), a program to identify plant transcription factors and transcriptional regulators and protein kinases, which currently provides both online and standalone versions with a hidden Markov model [107].

TEs were identified from the reference genome using RepeatMasker (http://www.repeatmasker.org/). TE density based on the length ratio of each bin within every chromosome was displayed as a Circos plot [48]. TEs annotated within gene set domains (including 2-kb upstream and downstream sequences) of DMR-related genes overlapping with DEGs were used for DNA methylation analysis. TEs were classified, and their distributions and mC contexts were analyzed.

### 4.6. Methylation Modification of Gene Expression

After the completion of independent methylome and transcriptome analyses, the correlation between methylation level and gene expression was progressively analyzed on four different levels. First, gene methylation level and gene expression densities were mapped onto the chromosomes of *P. mume*, and relationships between methylation levels and the expressions of promoters or gene coding regions, upstream (2-kb) and downstream (2-kb) regions, TSSs, and TESs were explored from a global perspective [48,90,108]. Second, CG, CHG, and CHH methylation modification patterns of DEGs (including promoters and 2-kb upstream and downstream regions) identified from the transcriptome data were analyzed [109,110]. Third, we attempted to relate the expression of DMR-related genes to different methylation modification patterns [111,112]. Finally, we identified the set of DMR-related genes that overlapped with DEGs, and subjected them to GO and KEGG enrichment analyses.

## 5. Conclusions

Flower color chimerism has since served as important material for landscaping application and genetic improvement. In our study, we detected the specific color substances, i.e., cyanidin 3,5-*O*-diglucoside, cyanidin 3-*O*-glucoside, and peonidin 3-*O*-glucoside, in red petal tissues of *P*. *mume* “Danban Tiaozhi”. Simultaneously, we investigated the molecular mechanism of chimeric flowers by using a comparative methylomic–transcriptomic approach. We mapped the first-ever generated methylomes of *P*. *mume*, and determined that gene expression was negatively correlated with methylcytosine level in general and uncovered significant epigenetic variation between WT and RT. We also detected DMRs and DMR-related genes between WT and RT, and concluded that many of these genes, including DEGs and transcription factor genes, are critical participants in the anthocyanin regulatory pathway. Importantly, some of the associated DEGs harbored TE insertions that were also modified by methylcytosine. It suggests that flower color chimerism in *P*. *mume* is induced by the DNA methylation of critical genes and TEs.

## Figures and Tables

**Table 1:** Methylation of C contexts mapping to the reference genome. mC/C: methylated cytosine to total cytosine.

	C Site	mC (mC/C)	CG	mCG (mCG/CG)	CHG	mCHG (mCHG/CHG)	CHH	mCHH (mCHH/CHH)
WT1	82368087	9302812 11.29%	8335974	3193016 38.30%	11664623	2275905 19.51%	62367490	3833891 6.14%
WT2	82368087	11706910 14.21%	8335974	3153580 37.83%	11664623	2396865 20.54%	62367490	6156465 9.87%
WT3	82368087	10982193 13.33%	8335974	3015038 36.16%	11664623	2258108 19.35%	62367490	5709047 9.15%
WT-average	82368087	10663972 12.94%	8335974	3120545 37.43%	11664623	2310293 19.80%	62367490	5233134 8.39%
RT1	82368087	11609228 14.09%	8335974	3086648 37.02%	11664623	2350640 20.15%	62367490	6171940 9.89%
RT2	82368087	11991003 14.55%	8335974	3170368 38.03%	11664623	2434099 20.86%	62367490	6386536 10.24%
RT3	82368087	12222792 14.83%	8335974	3411185 40.92%	11664623	2580335 22.12%	62367490	6231272 9.99%
RT-average	82368087	11941008 14.49%	8335974	3222734 38.66%	11664623	2455025 21.04%	62367490	6263249 10.04%
Average	82368087	11302490 13.72%	8335974	3171639 38.04%	11664623	2382659 20.42%	62367490	5748192 9.21%

“WT1”, “WT2” and “WT3” represent the white petal tissues; “RT1”, “RT2” and “RT3” represent the red petal tissues.

## Supplementary Materials

The following are available online at http://www.mdpi.com/1422-0067/19/8/2315/s1.
