# Supplementary material for: Substantial Epigenetic Variation Causing Flower Color Chimerism in the Ornamental Tree Prunus mume Revealed by Single Base Resolution Methylome Detection and Transcriptome Sequencing
Source: Int J Mol Sci. 2018 Aug 7;19(8):2315. doi: 10.3390/ijms19082315 (PMC6121637; doi:10.3390/ijms19082315)
Supplement: Supplementary file 1 [file ijms-19-02315-s001.zip › ijms-318958-suppl-proof done/suppl/ijms-318958-suppl-for proofreading.docx]

Supplementary Material

Substantial Epigenetic Variation Causing Flower Color Chimerism in the Ornamental Tree
*Prunus mume* Revealed by Single Base Resolution Methylome Detection and Transcriptome Sequencing

Kai-Feng Ma^1^, Qi-Xiang Zhang^1, 2*^, Tang-Ren Cheng^1^, Xiao-Lan Yan^3^, Hui-Tang Pan^1^, Jia Wang^1^

*** Correspondence:** Qixiang Zhang: [zqxbjfu@126.com](mailto:zqxbjfu@126.com)

Supplementary Figures


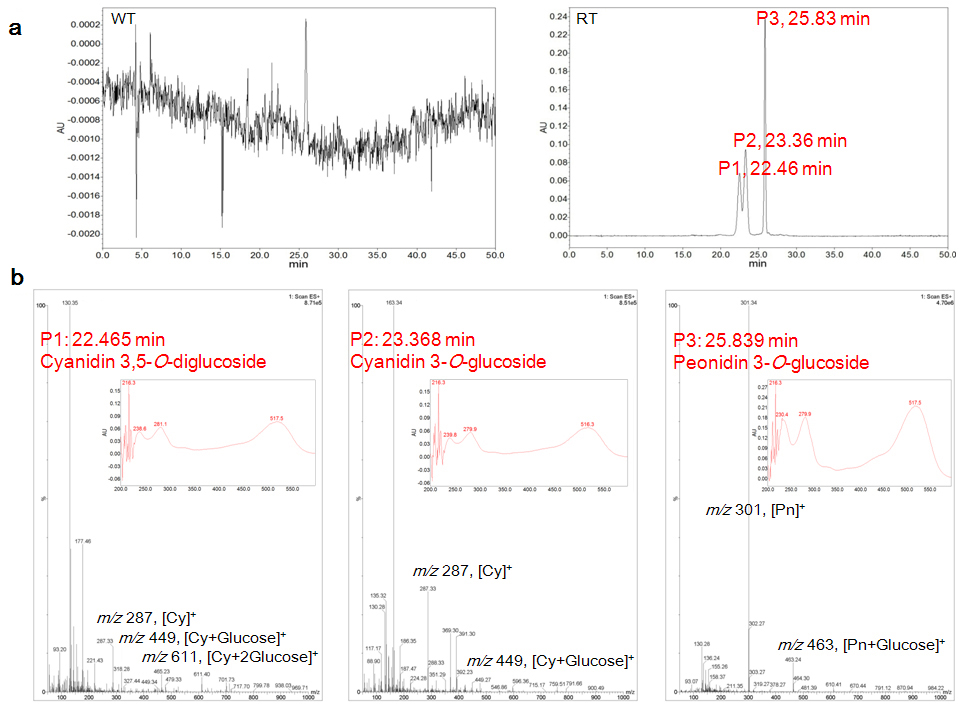


**Supplementary Figure S1. Detection of anthocyanidin within white petal tissues (WT) and red petal tissues (RT).** (**a**) Quantitative analysis on anthocyanidin using a high-performance liquid chromatography (HPLC) detection at 520 nm. (**b**) Qualitative investigation of each anthocyanin using mass spectra (MS) and HPLC with full band ultraviolet-visible scanning (200−600 nm).


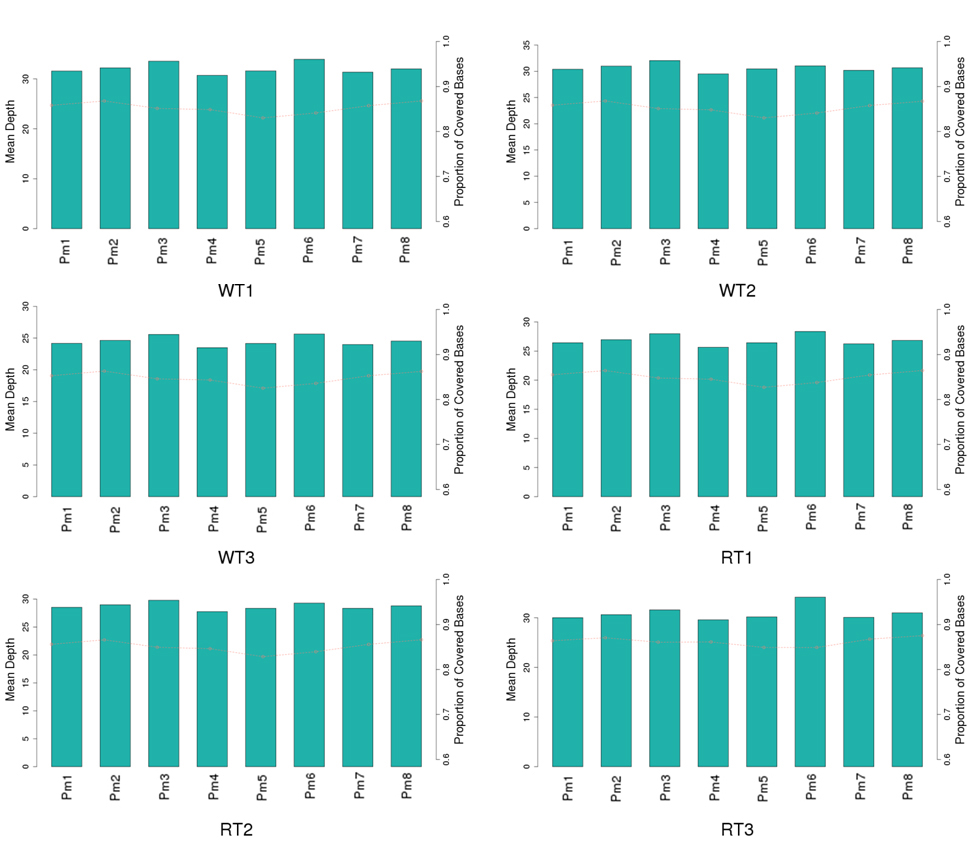


**Supplementary Figure S2. The depth and coverage of sequencing reads on each chromosome of the six samples of *Prunus mume*.** The *x*-coordinate indicates the name of each chromosome. The *y*-coordinate on the left and right indicate the average depth of the corresponding chromosome (column graphs), and proportion of reads covered by each chromosome (scatter plots).


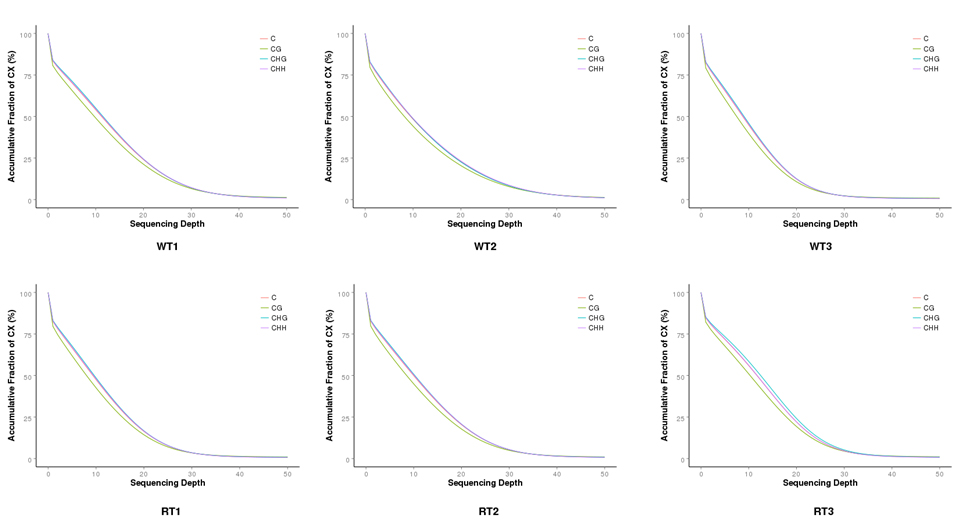


**Supplementary Figure S3. Distribution of cytosine-site coverage within each sample of *Prunus mume*.** The red, yellow–green, blue, and pink lines indicate C, CG, CHG, and CHH-contexts, respectively. The *x*-coordinate and *y*-coordinate indicate the sequencing depth and accumulative fraction of C-contexts, respectively.


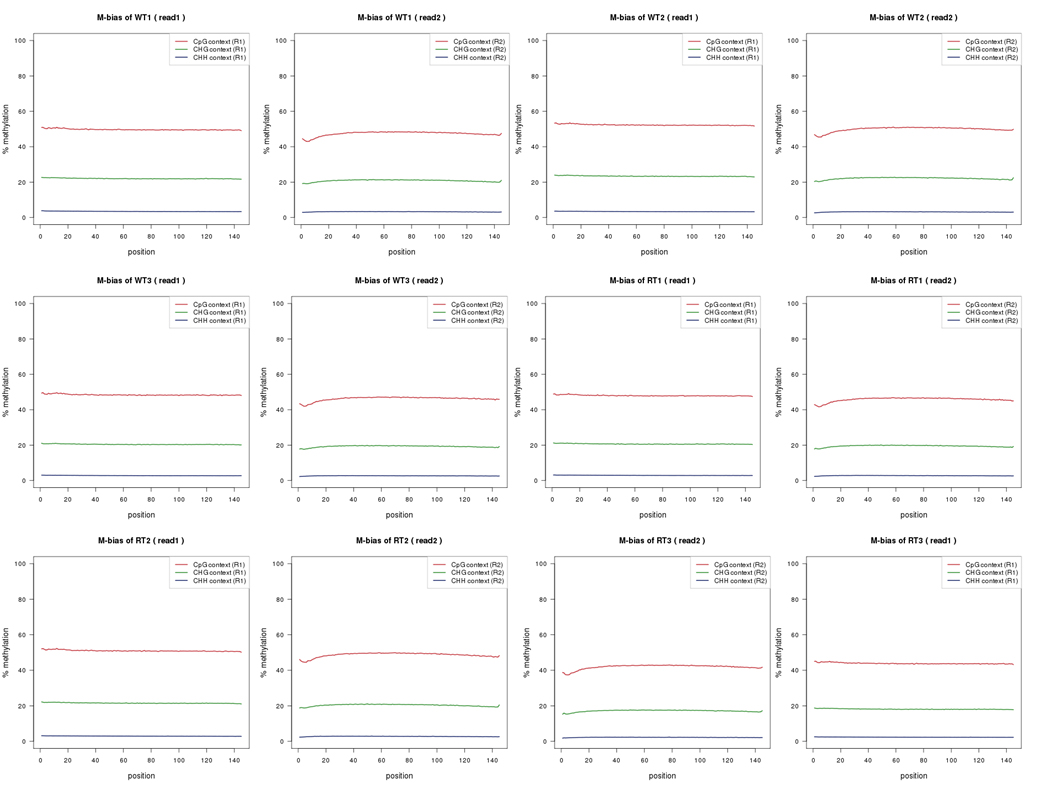


**Supplementary Figure S4. M-bias plot of the methylation level assessment of *Prunus mume* genome**. The *x*-coordinate and *y*-coordinate indicate the position of sequencing reads and DNA methylation level, respectively.


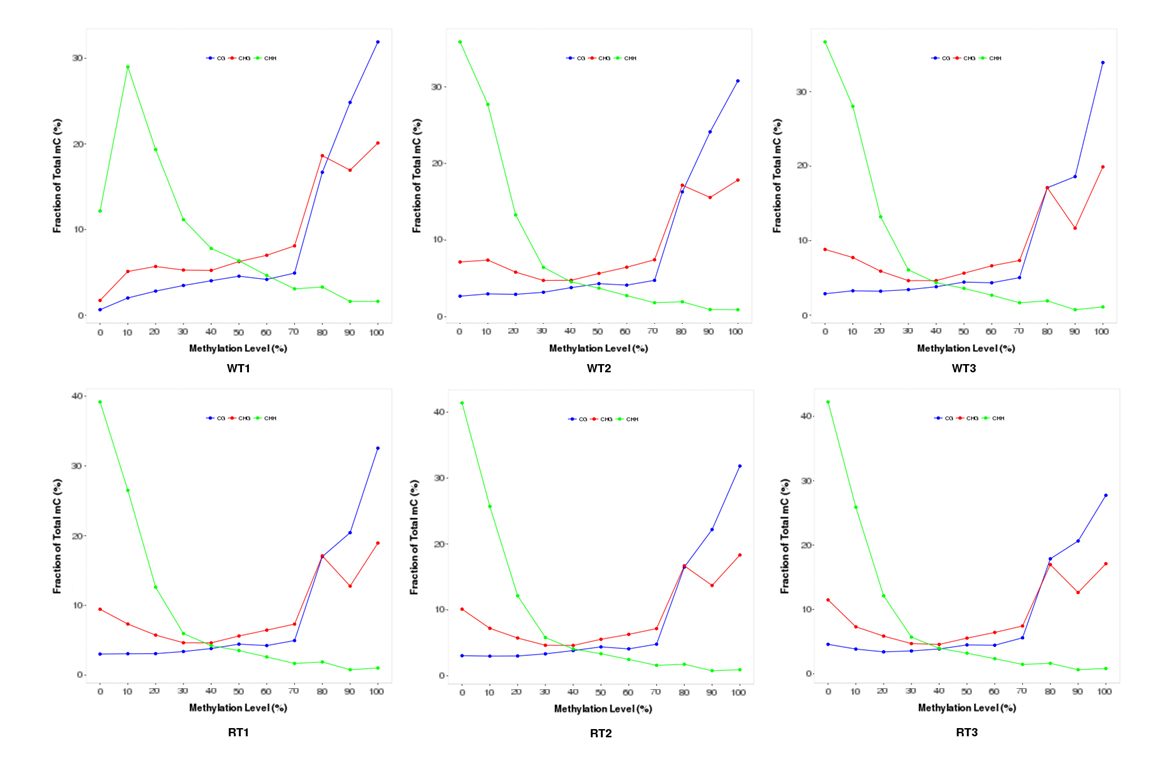


**Supplementary Figure S5. Distribution of mC-contexts levels.** The blue, red, and green broken lines indicate mCG, mCHG, and mCHHcontexts. The*x*-coordinate indicates methylation level, and the *y*-coordinate indicates the fraction of each mC-contexts site’s corresponding methylation level to the total site of each mC-context. .


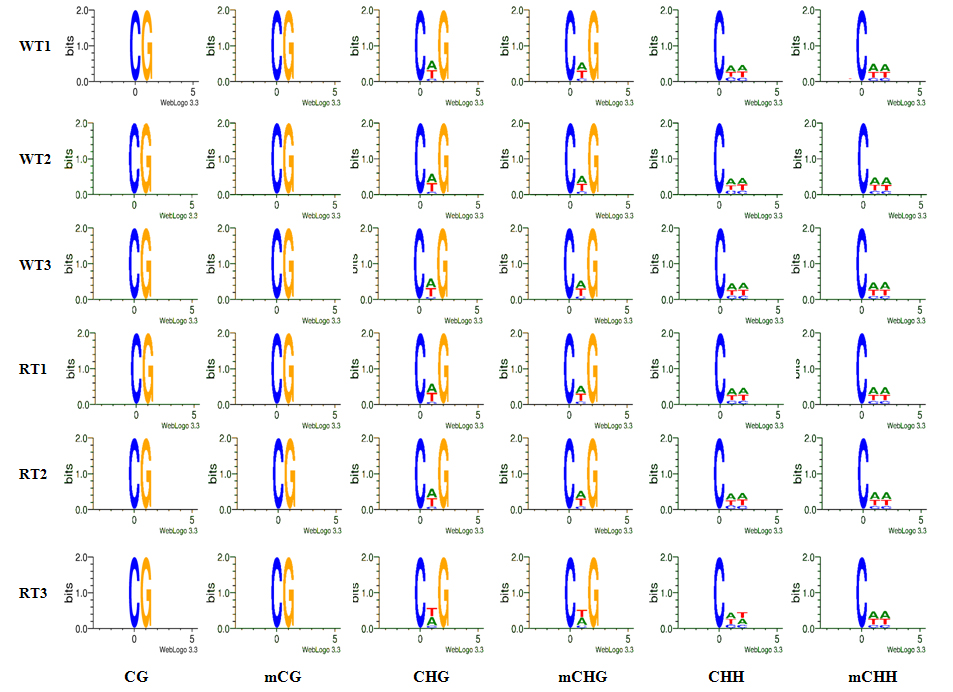


**Supplementary Figure S6. Weblogo analysis on basesaround mC in different sequence contexts of *Prunus mume* genomes.** ‘WT1’, ‘WT2’, and ‘WT3’ represent the white petal tissues 1, 2, and 3, respectively.‘RT1’, ‘RT2’, and ‘RT3’ represent the red petal tissues 1, 2, and 3, respectively. The CG, CHG, and CHH represent all of the CG, CHG, and CHH-contexts, respectively.mCG, mCHG, and mCHH represent mCG, mCHG, and mCHH-contexts, respectively.The*x*-coordinate indicatesthe base position, and *y*-coordinate indicates the base enrichment degree. The position ofC-site was defined as 0.


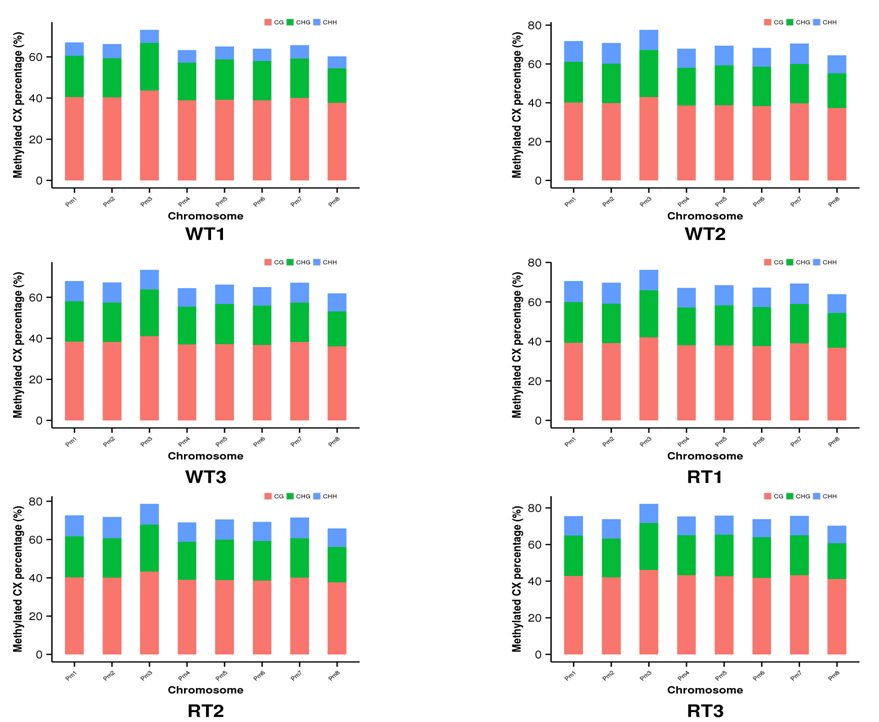


**Supplementary Figure S7. The percentage of each mC-context to total methylation sites on each chromosome of *Prunus mume*.**‘WT1’, ‘WT2’, and ‘WT3’ represent white petal tissues 1, 2, and 3, respectively. ‘RT1’, ‘RT2’, and ‘RT3’ represent red petal tissues 1, 2, and 3, respectively.The *x*-coordinate indicates the chromosome and the*y*-coordinate indicates the percentage of mC-contexts. CG (red), CHG (green), and CHH (blue) represent the mCG, mCHG, and mCHHcontexts, respectively.


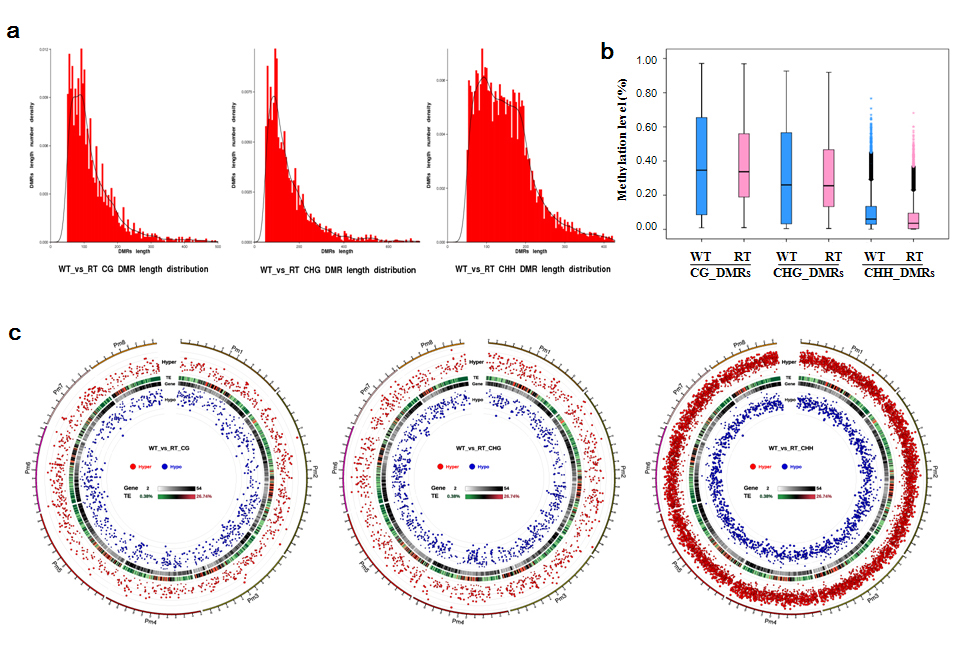


**Supplementary Figure S8. Analysis on differential methylated regions (DMRs).** (**a**) Frequency distribution of DMRs’ length. The *x*-coordinate indicates the length of the DMRs, the*y*-coordinate indicatesthe density of the DMRs. ‘WT_vs_RT’represents white petal tissues vs. red petal tissues. (**b**) Box plot of methylation level of DMRs. ‘WT’ and ‘RT’representwhite petal tissues and red petal tissues, respectively. The *x*-coordinate indicatesa sample name and different sequence of DMRs, the*y*-coordinate indicatesthe methylation level. (**c**) Circos plots of CG, CHG, and CHH DMRs, and density plot of transposable elements (TEs) and genes (Gene) on each chromosome of *Prunus mume*. Track order (outside–in): scatter plot of hypermethylation (Hyper; red), density plot of TEs (TE), density plot of genes (Gene), and scatter plot of hypermethylation (Hypo; blue).


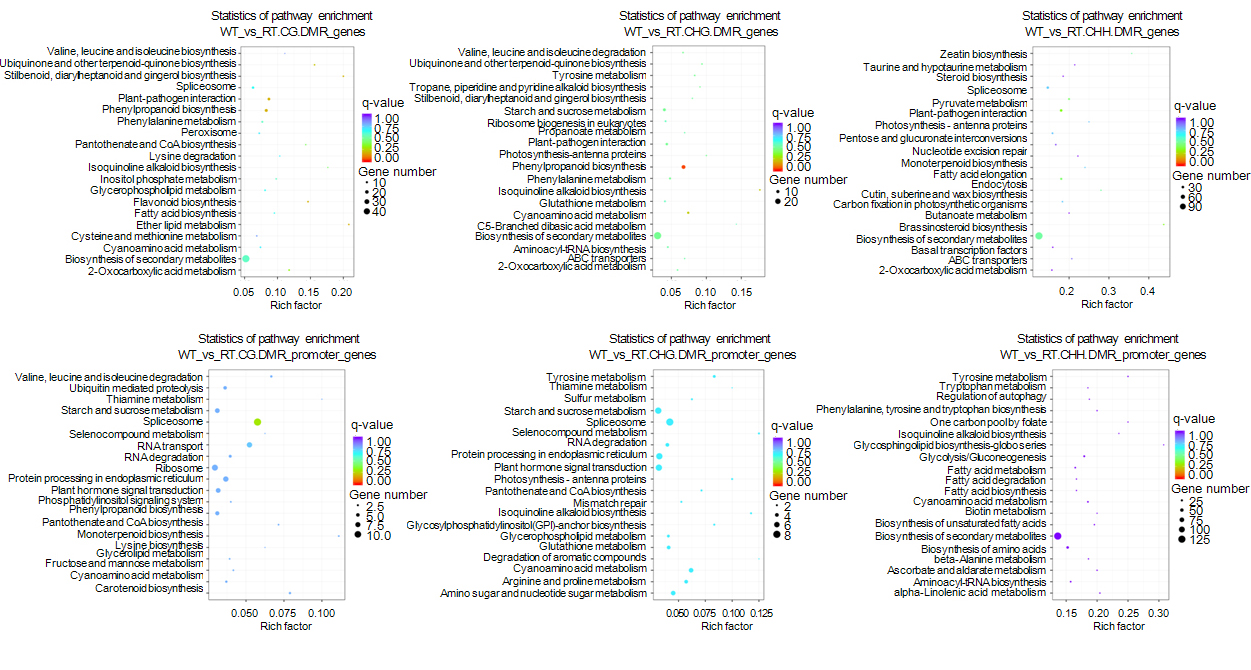


**Supplementary Figure S9. KEGG pathway enrichment of differentially methylated region (DMR)-related genes.** WT, white petal tissues; RT, red petal tissues. CG, CHG, and CHH DMR_genes and DMR_promoter_genes refer to CG, CHG, and CHH DMR-related genes anchored within genebody and promoter domains, respectively.


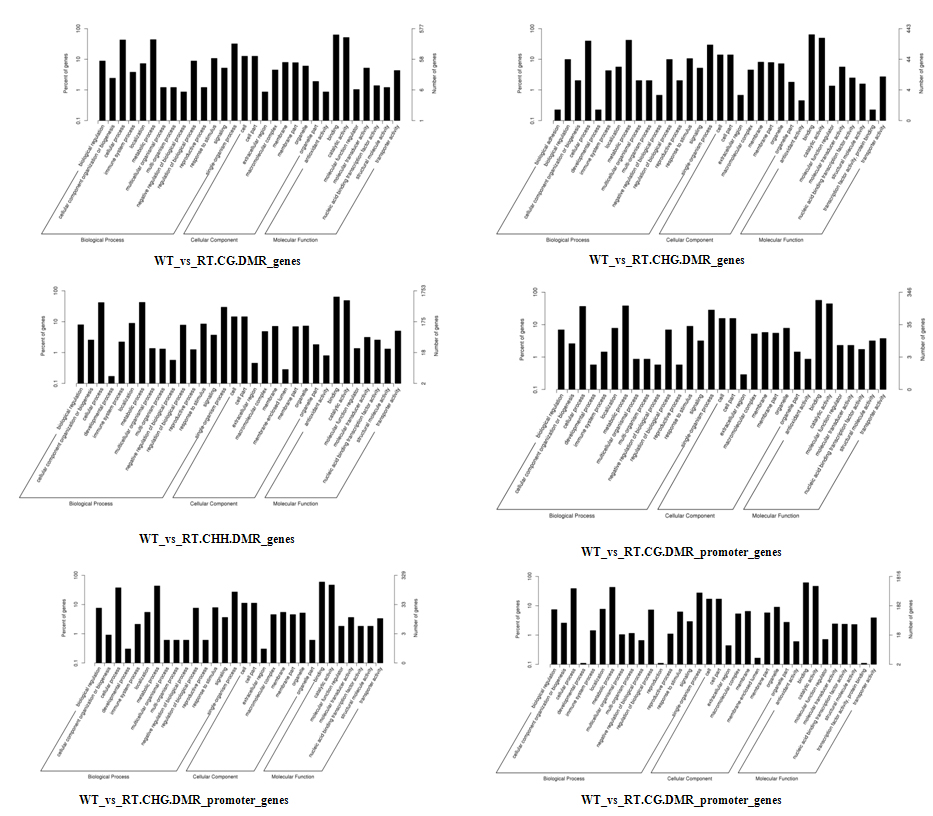


**Supplementary Figure S10. Gene ontology pathway enrichment of the differential methylated region (DMR)-related genes.**‘WT_vs_RT’represents white petal tissues vs. red petal tissues. CG, CHG, and CHH DMR_genes indicate CG, CHG, and CHH DMRs-related genes were anchored within genebody domains. CG, CHG, and CHH DMR_promoter_genes indicate CG, CHG, and CHH DMRs-related genes were anchored within promoter domains.


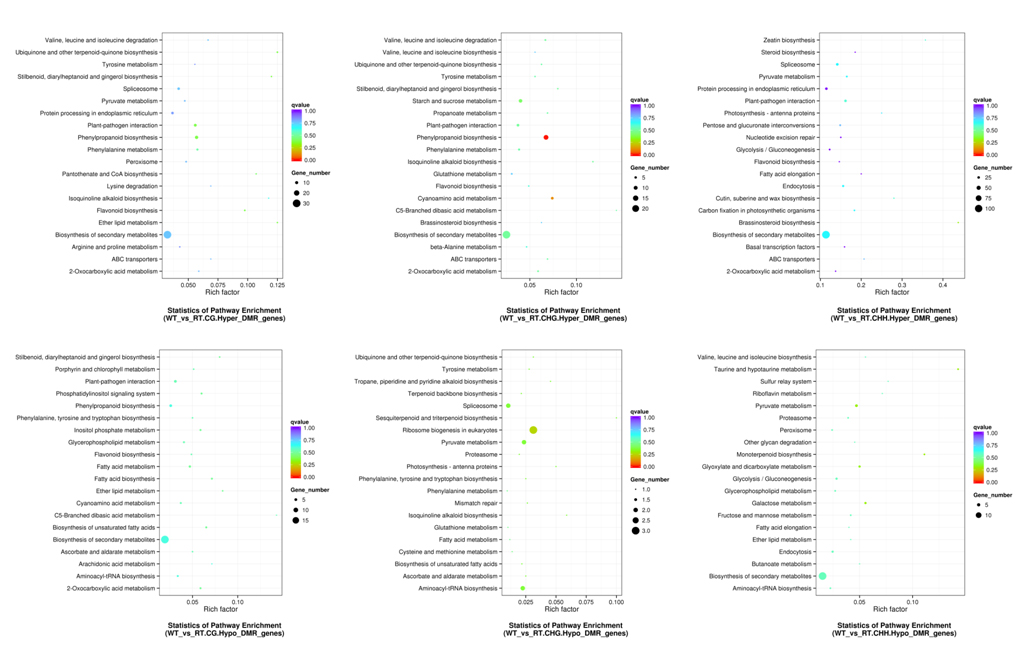


**Supplementary Figure S11. KEGG pathway enrichment of the differential methylated region (DMR)-related genes.** ‘WT_vs_RT’represents white petal tissues vs. red petal tissues.These DMRs-related genes were anchored within genebody domains.


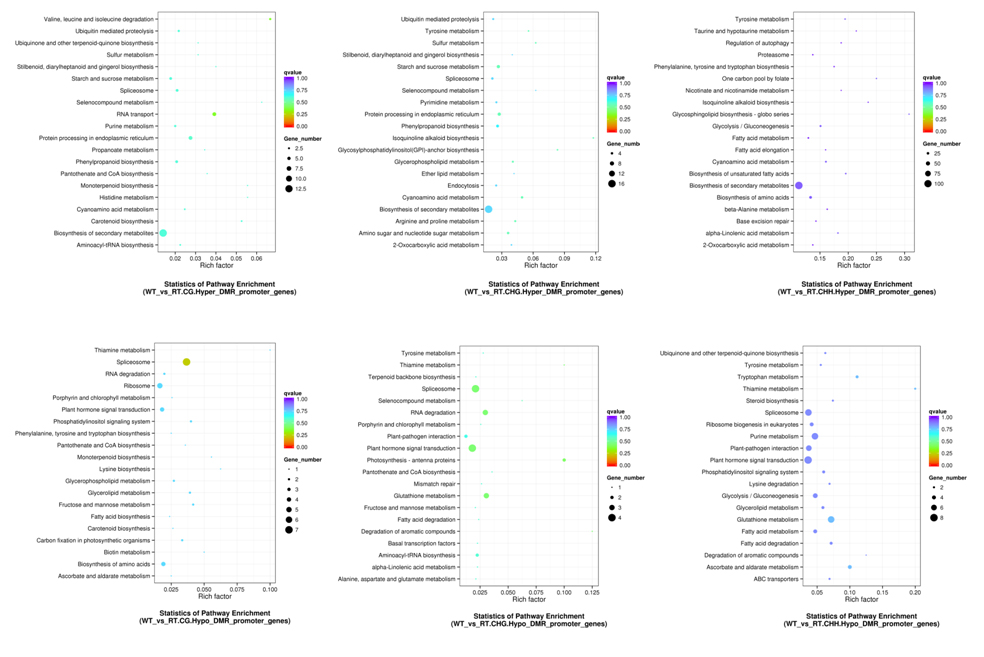


**Supplementary Figure S12. KEGG pathway enrichment of the differential methylated region (DMR)-related genes.** ‘WT_vs_RT’represents white petal tissues vs. red petal tissues.These DMR-related genes were anchored within promoter domains.


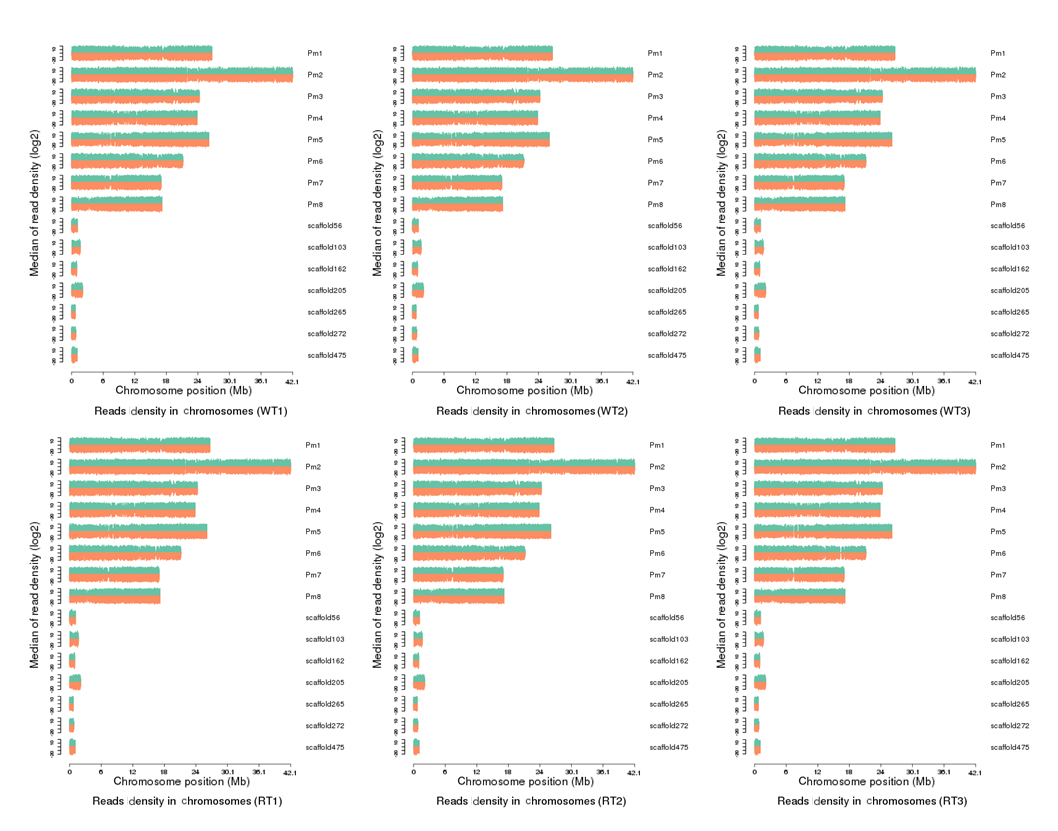


**Supplementary Figure S13. Density distribution of the sequencing reads on the chromosome of *Prunus mume*transcriptomes.** ‘WT1’, ‘WT2’, and ‘WT3’ represent white petal tissues 1, 2, and 3, respectively. ‘RT1’, ‘RT2’, and ‘RT3’ represent red petal tissues 1, 2, and 3, respectively.The *x*-coordinate indicates reads chromosome position, and the *y*-coordinate indicates chromosome name.


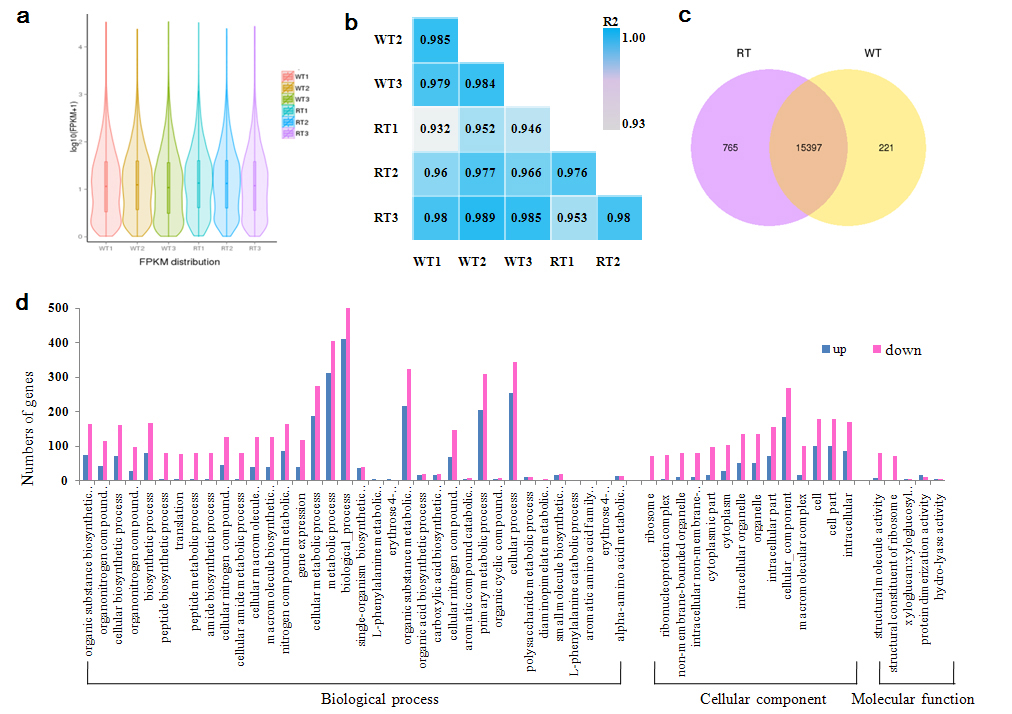


**Supplementary Figure S14. Analysis on gene expression.** (**a**) Violin plots of gene expression level. The *x*-coordinate indicates samples name, and the*y*-coordinate indicates gene expression level. ‘WT1’, ‘WT2’, and ‘WT3’ represent white petal tissues 1, 2, and 3, respectively. ‘RT1’, ‘RT2’, and ‘RT3’ represent red petal tissues 1, 2, and 3, respectively. (**b**) Linear correlations between gene expression of each two of the six samples. ‘WT1’, ‘WT2’, and ‘WT3’ represent white petal tissues 1, 2, and 3, respectively. ‘RT1’, ‘RT2’, and ‘RT3’ represent red petal tissues 1, 2, and 3, respectively. (**c**) Specific and common expression between white petal tissues (WT) and red petal tissues (RT). (**d**) Gene ontology pathway enrichment of differentially expressed genes (DEGs).


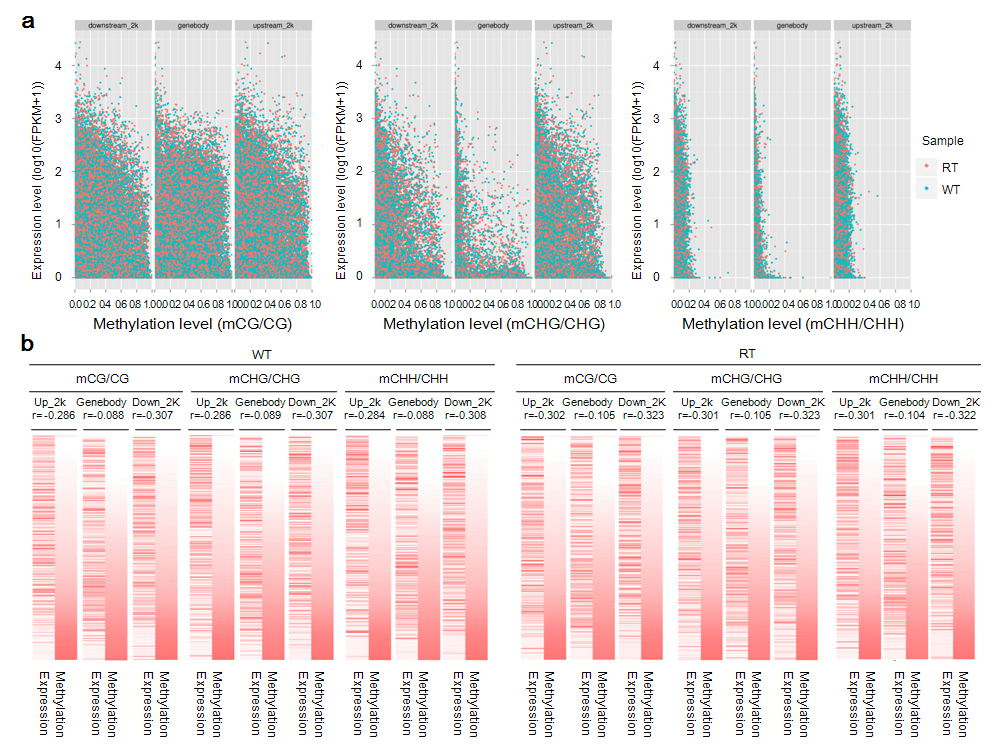


**Supplementary Figure S15. Analysis of gene methylation by sequence context (mCG, mCHG, and mCHH) and genome-wide gene expression**. WT and RT indicate white petal tissues and red petal tissues, respectively. (**a**) Scatter plots of methylation and gene expression levels within gene-bodies (including 2-kb upstream and downstream regions). The *x* and *y*-axes indicate DNA methylation levels and gene expression levels, respectively. (**b**) Heat maps illustrating linear correlations between mCG, mCHG, and mCHH methylation levels and gene expression levels.


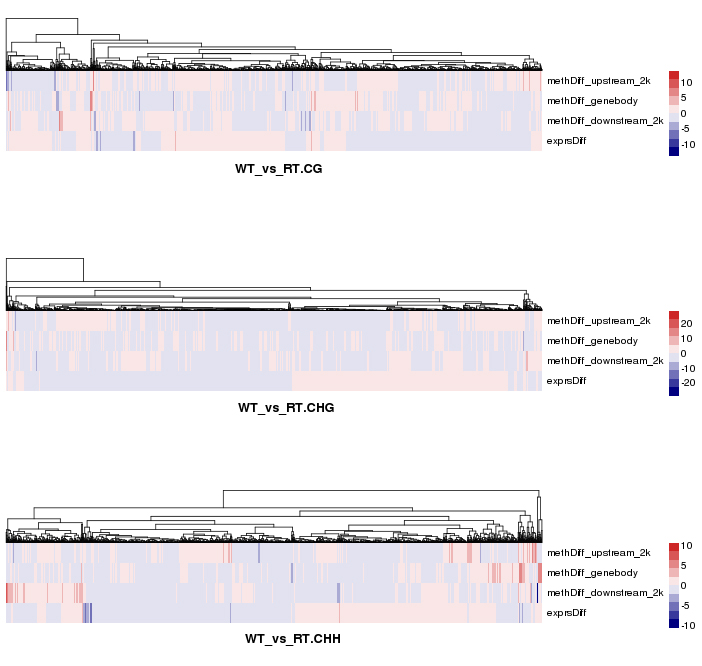


**SupplementaryFigureS16**. **The hierarchical clustering analysis on the different expression and methylation levels of differentially expressed genes (DEGs)**. The ‘WT_vs_RT.CG’, ‘WT_vs_RT.CHG’, and ‘WT_vs_RT.CHH’ represent CG, CHG, and CHHcontexts of white petal tissues vs. red petal tissues. The ‘expresDiff’ indicates the expression difference of DMRs. The ‘methDiff_gene-body’, ‘methDiff_upstream_2k’, and ‘methDiff_downstream_2k’ represent the methylation difference of genebody domain 2-kb upstream of gene and 2-kb downstream of the gene domains, respectively.


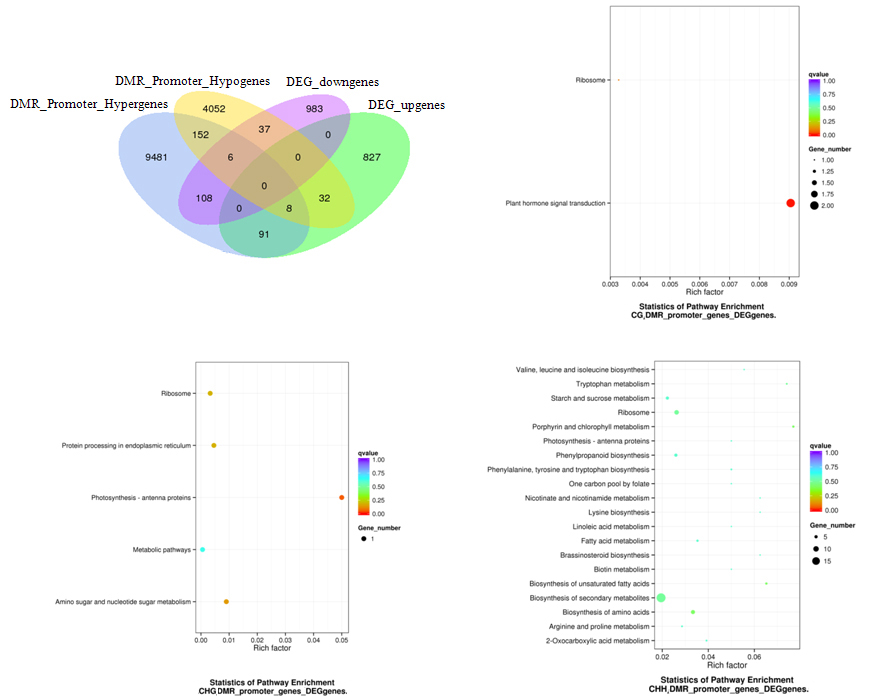


**Supplementary Figure S17. Detection and KEGG pathway enrichment of differentially methylated region (DMR)-related differentially expressed genes (DEGs).** These DEGs are anchored within promoters domains by mC-context DMRs. ‘DMR_Promoter_Hypergenes’, and ‘DMR_Promoter_Hypogenes’ indicate hypermethylated and hypomethylated DMRs-related genes, respectively. ‘DEG_downgenes’ and ‘DEG_upgenes’ indicate downregulated and upregulated DEGs, respectively. The ‘CG.DMR_promoter_genes’, ‘CHG.DMR_promoter_genes’, and ‘CHH.DMR_promoter_genes’ represent CG, CHG, and CHH DMR-related genes, respectively.


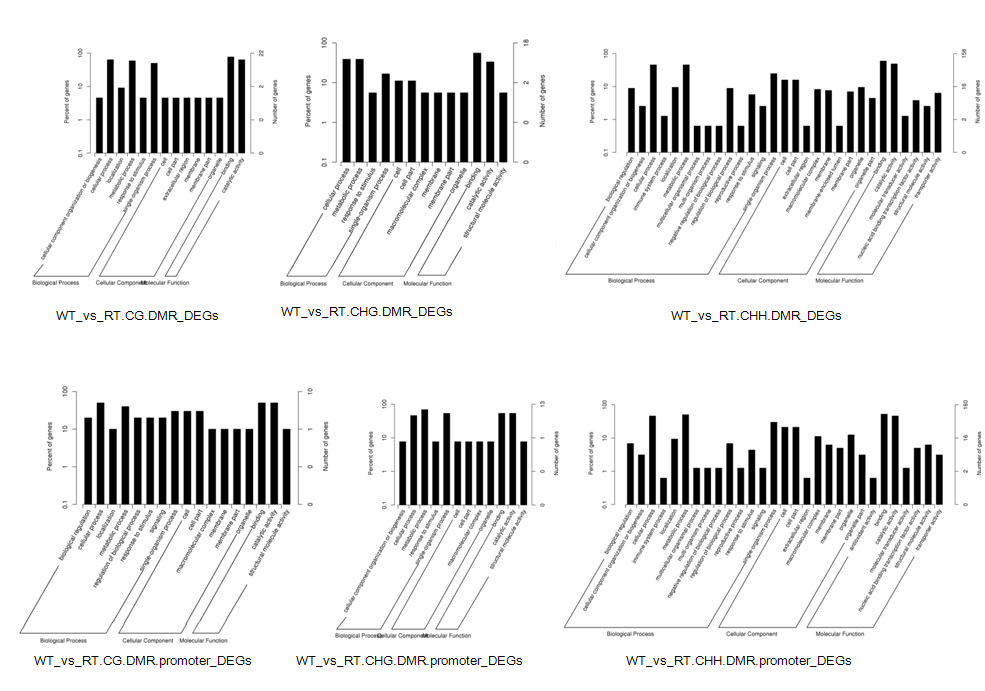


**Supplementary Figure S18. Gene ontology pathway enrichment of the differential methylated region (DMR)-related differentially expressed genes (DEGs).**


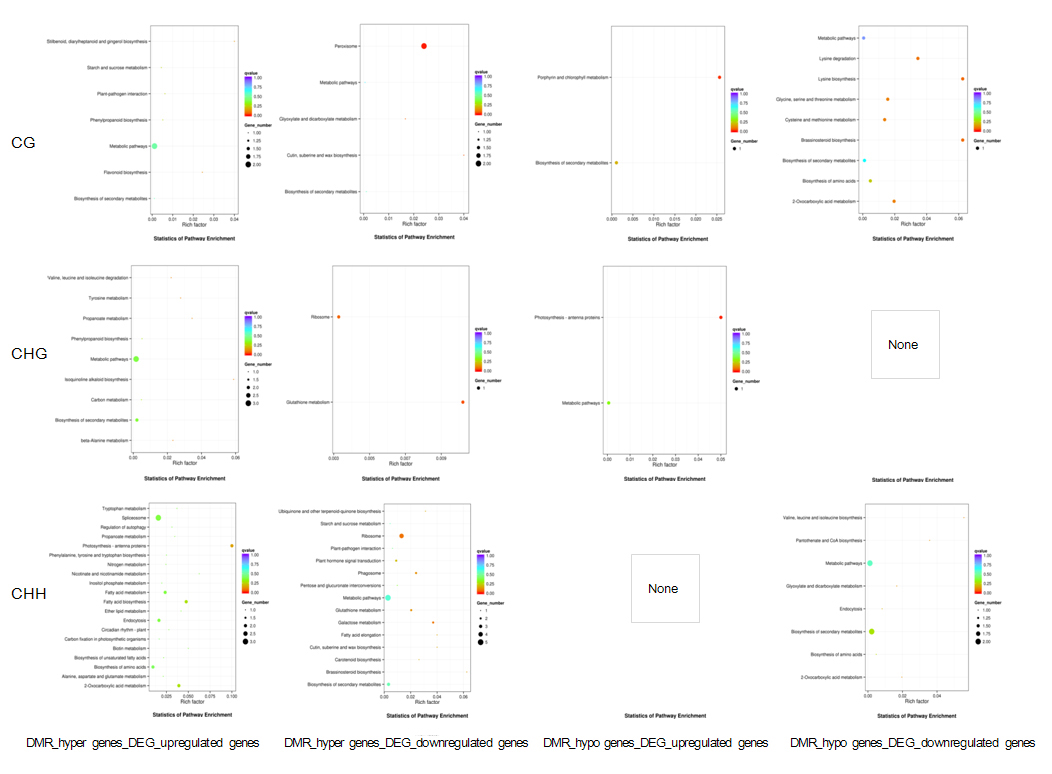


**Supplementary Figure S19. KEGG pathway enrichment of the differential methylated region (DMR)-related differentially expressed genes (DEGs)**. These DEGs were anchored within genebody domains by mC-context DMRs.


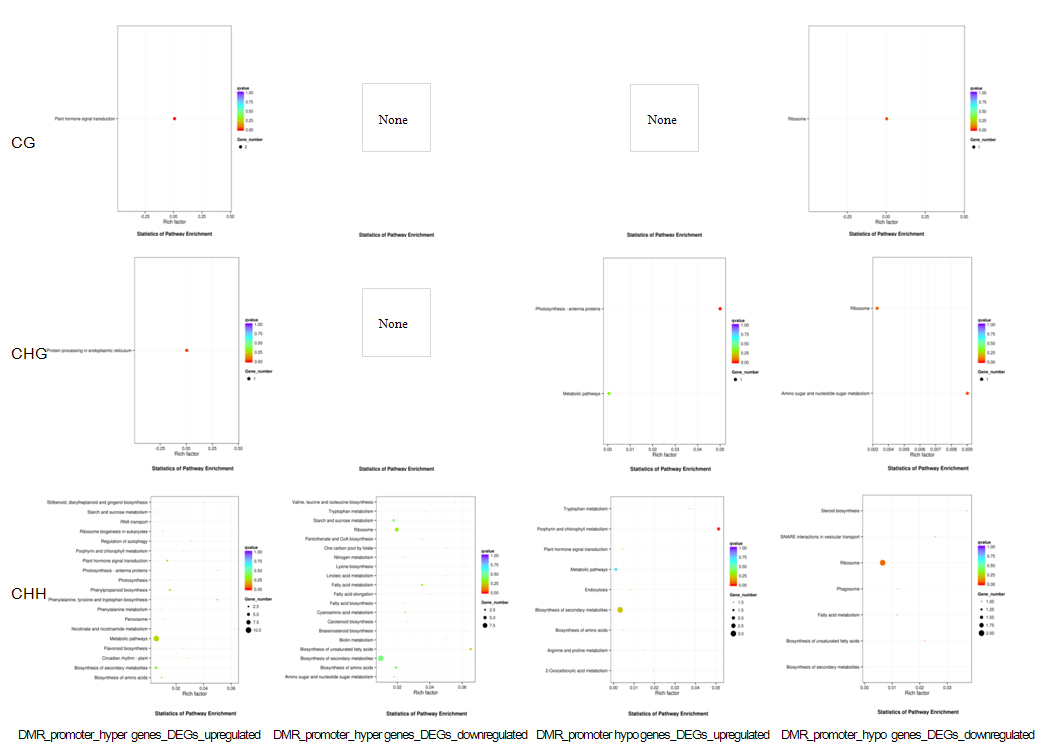


**Supplementary Figure S20. KEGG pathway enrichment of the differential methylated region (DMR)-related differentially expressed genes (DEGs).** These DEGs were anchored within promoter domains by mC-context DMRs.

Supplementary Tables

**Supplementary Table S1**. Sequencing and mapping data of BS-seq for the six samples.

| Sample name | Raw reads | Raw bases  (G) | Clean reads | Clean bases  (G) | Cleanratio  (%) | Q20  (%) | Q30  (%) | GC content  (%) | BS conversion rate  (%) | Total reads | Mapped reads | Mapping rate  (%) | Duplication rate  (%) |
| --- | --- | --- | --- | --- | --- | --- | --- | --- | --- | --- | --- | --- | --- |
| WT1 | 52288362 | 15.69 | 50903918 | 14.57 | 92.86 | 97.67 | 93.06 | 21.54 | 99.108 | 50903918 | 29987498 | 58.91 | 13.53 |
| WT2 | 49964869 | 14.99 | 48881795 | 14.00 | 93.40 | 97.89 | 93.59 | 21.4 | 99.802 | 48881795 | 28361217 | 58.02 | 13.74 |
| WT3 | 40554528 | 12.17 | 40027617 | 11.62 | 95.48 | 98.29 | 94.97 | 21.31 | 99.812 | 40027617 | 24068606 | 60.13 | 19.09 |
| RT1 | 44826723 | 13.45 | 44129589 | 12.77 | 94.94 | 98.16 | 94.6 | 21.37 | 99.838 | 44129589 | 26354190 | 59.72 | 18.56 |
| RT2 | 44738504 | 13.42 | 43909246 | 12.44 | 92.70 | 97.61 | 92.58 | 21.41 | 99.84 | 43909246 | 26016228 | 59.25 | 11.13 |
| RT3 | 48581198 | 14.57 | 47408351 | 13.6 | 93.34 | 97.68 | 93.11 | 21.45 | 99.862 | 47408351 | 28791091 | 60.73 | 13.26 |

‘WT1’, ‘WT2’ and ‘WT3’ represent the white petal tissues; ‘RT1’, ‘RT2’ and ‘RT3’ represent the red petal tissues.

**Supplementary Table S2.** C-site coverage and percentage of methylation levels of each sample.

| Sample | Mean of C coverage | C number  (Mb) | CG  (Mb) | CHG  (Mb) | CHH  (Mb) | Mean of  mC/C  (%) | Mean of  mCG/CG  (%) | Mean of  mCHG/CHG  (%) | Mean of  mCHH/CHH  (%) |
| --- | --- | --- | --- | --- | --- | --- | --- | --- | --- |
| WT1 | 13.8 | 1137.8 | 117.6 | 164.9 | 855.4 | 10.69 | 48.7 | 21.51 | 3.38 |
| WT2 | 12.9 | 1062.6 | 108 | 151.9 | 802.7 | 10.95 | 51.24 | 22.82 | 3.28 |
| WT3 | 10.5 | 862.9 | 87.5 | 125.4 | 650 | 9.77 | 47.44 | 19.93 | 2.74 |
| RT1 | 11.5 | 948 | 97 | 137.9 | 713.2 | 9.88 | 47.03 | 20.14 | 2.85 |
| RT2 | 12.2 | 1006.3 | 101.2 | 145.7 | 759.5 | 10.24 | 49.99 | 21.04 | 2.87 |
| RT3 | 13.6 | 1118.1 | 115.6 | 166.8 | 835.6 | 8.8 | 43.01 | 17.71 | 2.29 |

‘WT1’, ‘WT2’ and ‘WT3’ represent the white petal tissues; ‘RT1’, ‘RT2’ and ‘RT3’ represent the red petal tissues.

**Supplementary Table S3.** Quality control and mapping statistics of sequencing data generated from RNA-seq.

| Sample  name | Raw  reads | Clean  reads | Clean  bases  (G) | Q20  (%) | Q30  (%) | GC  content  (%) | Total  reads | Total  mapped | Multiple  mapped | Uniquely  mapped | Reads  map to ‘+’ | Reads  map to ‘-’ |
| --- | --- | --- | --- | --- | --- | --- | --- | --- | --- | --- | --- | --- |
| WT1 | 57104966 | 55656090 | 8.35 | 94.8 | 87.98 | 45.28 | 55656090 | 49062666  (88.15%) | 1479313  (2.66%) | 47583353  (85.5%) | 23713159  (42.61%) | 23870194  (42.89%) |
| RT1 | 51734984 | 50316828 | 7.55 | 94.74 | 87.83 | 45.22 | 50316828 | 44461055  (88.36%) | 1349407  (2.68%) | 43111648  (85.68%) | 21486594  (42.7%) | 21625054  (42.98%) |
| WT2 | 50649152 | 49185526 | 7.38 | 94.64 | 87.65 | 45.26 | 49185526 | 43028462  (87.48%) | 1364423  (2.77%) | 41664039  (84.71%) | 20762752  (42.21%) | 20901287  (42.49%) |
| RT2 | 49429354 | 47958828 | 7.19 | 94.63 | 87.59 | 45.14 | 47958828 | 42149022  (87.89%) | 1237769  (2.58%) | 40911253  (85.3%) | 20383369  (42.5%) | 20527884  (42.8%) |
| WT3 | 45379114 | 44202732 | 6.63 | 94.88 | 88.13 | 45.37 | 44202732 | 38737776  (87.64%) | 1248586  (2.82%) | 37489190  (84.81%) | 18677892  (42.26%) | 18811298  (42.56%) |
| RT3 | 50160666 | 48750242 | 7.31 | 94.9 | 88.12 | 45.32 | 48750242 | 42957271  (88.12%) | 1327651  (2.72%) | 41629620  (85.39%) | 20744427  (42.55%) | 20885193  (42.84%) |

‘WT1’, ‘WT2’ and ‘WT3’ represent the white petal tissues; ‘RT1’, ‘RT2’ and ‘RT3’ represent the red petal tissues.

**Supplementary Table S4**. Statistics of transcription factors detected from the transcriptomes.

| TF_ID | Family | Type | Hyperlink | Description |
| --- | --- | --- | --- | --- |
| Pm000163_0 | WRKY | transcription factor | http://planttfdb.cbi.pku.edu.cn/family.php?fam=WRKY | WRK33_ARATH Probable WRKY transcription factor 33 OS=Arabidopsis thaliana GN=WRKY33 PE=1 SV=2 |
| Pm000232_0 | AP2-EREBP | transcription factor | -- | PTI6_SOLLC Pathogenesis-related genes transcriptional activator PTI6 OS=Solanum lycopersicum GN=PTI6 PE=2 SV=1 |
| Pm000251_0 | bZIP | transcription factor | http://planttfdb.cbi.pku.edu.cn/family.php?fam=bZIP | RF2B_ORYSJ Transcription factor RF2b OS=Oryza sativa subsp. japonica GN=RF2b PE=1 SV=1 |
| Pm000264_0 | OFP | transcription factor | -- | -- |
| Pm000304_0 | WRKY | transcription factor | http://planttfdb.cbi.pku.edu.cn/family.php?fam=WRKY | WRKY2_ARATH Probable WRKY transcription factor 2 OS=Arabidopsis thaliana GN=WRKY2 PE=2 SV=1 |
| Pm000447_0 | bHLH | transcription factor | http://planttfdb.cbi.pku.edu.cn/family.php?fam=bHLH | BH122_ARATH Transcription factor bHLH122 OS=Arabidopsis thaliana GN=BHLH122 PE=1 SV=1 |
| Pm000539_0 | C2H2 | transcription factor | http://planttfdb.cbi.pku.edu.cn/family.php?fam=C2H2 | -- |
| Pm000578_0 | C3H | transcription factor | http://planttfdb.cbi.pku.edu.cn/family.php?fam=C3H | C3H66_ARATH Zinc finger CCCH domain-containing protein 66 OS=Arabidopsis thaliana GN=At5g58620 PE=2 SV=1 |
| Pm000604_0 | GNAT | transcriptional regulator | -- | -- |
| Pm000614_0 | TRAF | transcriptional regulator | -- | ETO1_ARATH Ethylene-overproduction protein 1 OS=Arabidopsis thaliana GN=ETO1 PE=1 SV=2 |
| Pm000717_0 | GRAS | transcription factor | http://planttfdb.cbi.pku.edu.cn/family.php?fam=GRAS | SCL14_ARATH Scarecrow-like protein 14 OS=Arabidopsis thaliana GN=SCL14 PE=2 SV=2 |
| Pm000718_0 | GRAS | transcription factor | http://planttfdb.cbi.pku.edu.cn/family.php?fam=GRAS | SCL14_ARATH Scarecrow-like protein 14 OS=Arabidopsis thaliana GN=SCL14 PE=2 SV=2 |
| Pm000974_0 | AP2-EREBP | transcription factor | -- | RAP27_ARATH Ethylene-responsive transcription factor RAP2-7 OS=Arabidopsis thaliana GN=RAP2-7 PE=2 SV=2 |
| Pm001160_0 | C2H2 | transcription factor | http://planttfdb.cbi.pku.edu.cn/family.php?fam=C2H2 | ZFP1_WHEAT Zinc finger protein 1 OS=Triticum aestivum PE=2 SV=1 |
| Pm001211_0 | MADS | transcription factor | -- | -- |
| Pm002052_0 | AP2-EREBP | transcription factor | -- | RAP24_ARATH Ethylene-responsive transcription factor RAP2-4 OS=Arabidopsis thaliana GN=RAP2-4 PE=1 SV=1 |
| Pm002327_0 | HB | transcription factor | -- | HAT22_ARATH Homeobox-leucine zipper protein HAT22 OS=Arabidopsis thaliana GN=HAT22 PE=1 SV=1 |
| Pm002393_0 | TAZ | transcription factor | -- | BT4_ARATH BTB/POZ and TAZ domain-containing protein 4 OS=Arabidopsis thaliana GN=BT4 PE=1 SV=1 |
| Pm002404_0 | LOB | transcription factor | -- | LBD38_ARATH LOB domain-containing protein 38 OS=Arabidopsis thaliana GN=LBD38 PE=2 SV=1 |
| Pm002485_0 | WRKY | transcription factor | http://planttfdb.cbi.pku.edu.cn/family.php?fam=WRKY | WRKY7_ARATH Probable WRKY transcription factor 7 OS=Arabidopsis thaliana GN=WRKY7 PE=1 SV=1 |
| Pm002492_0 | AP2-EREBP | transcription factor | -- | AP2_ARATH Floral homeotic protein APETALA 2 OS=Arabidopsis thaliana GN=AP2 PE=1 SV=1 |
| Pm002536_0 | MYB | transcription factor | http://planttfdb.cbi.pku.edu.cn/family.php?fam=MYB | MY1R1_SOLTU Transcription factor MYB1R1 OS=Solanum tuberosum PE=2 SV=1 |
| Pm002826_0 | WRKY | transcription factor | http://planttfdb.cbi.pku.edu.cn/family.php?fam=WRKY | WRK44_ARATH WRKY transcription factor 44 OS=Arabidopsis thaliana GN=WRKY44 PE=1 SV=2 |
| Pm002827_0 | Pseudo ARR-B | transcriptional regulator | -- | APRR7_ARATH Two-component response regulator-like APRR7 OS=Arabidopsis thaliana GN=APRR7 PE=2 SV=1 |
| Pm002840_0 | MYB | transcription factor | http://planttfdb.cbi.pku.edu.cn/family.php?fam=MYB | ASG4_ARATH Transcription factor ASG4 OS=Arabidopsis thaliana GN=ASG4 PE=2 SV=1 |
| Pm003368_0 | CCAAT | transcription factor | -- | NFYC9_ARATH Nuclear transcription factor Y subunit C-9 OS=Arabidopsis thaliana GN=NFYC9 PE=2 SV=1 |
| Pm003418_0 | CCAAT | transcription factor | -- | H2AX_CICAR Histone H2AX OS=Cicer arietinum GN=HIS2A PE=2 SV=1 |
| Pm003477_0 | GeBP | transcription factor | http://planttfdb.cbi.pku.edu.cn/family.php?fam=GeBP | -- |
| Pm003502_0 | Trihelix | transcription factor | http://planttfdb.cbi.pku.edu.cn/family.php?fam=Trihelix | GTL2_ARATH Trihelix transcription factor GTL2 OS=Arabidopsis thaliana GN=At5g28300 PE=2 SV=1 |
| Pm003525_0 | bHLH | transcription factor | http://planttfdb.cbi.pku.edu.cn/family.php?fam=bHLH | UNE12_ARATH Transcription factor UNE12 OS=Arabidopsis thaliana GN=UNE12 PE=2 SV=2 |
| Pm003529_0 | AUX/IAA | transcriptional regulator | -- | AX22B_VIGRR Auxin-induced protein 22B OS=Vigna radiata var. radiata GN=AUX22B PE=2 SV=1 |
| Pm003530_0 | AUX/IAA | transcriptional regulator | -- | IAA16_ARATH Auxin-responsive protein IAA16 OS=Arabidopsis thaliana GN=IAA16 PE=2 SV=1 |
| Pm003647_0 | MYB | transcription factor | http://planttfdb.cbi.pku.edu.cn/family.php?fam=MYB | VDE_SPIOL Violaxanthin de-epoxidase, chloroplastic OS=Spinacia oleracea GN=VDE1 PE=1 SV=2 |
| Pm003827_0 | ARF | transcription factor | http://planttfdb.cbi.pku.edu.cn/family.php?fam=ARF | ARFA_ARATH Auxin response factor 1 OS=Arabidopsis thaliana GN=ARF1 PE=1 SV=2 |
| Pm003895_0 | ABI3VP1 | transcription factor | -- | VRN1_ARATH B3 domain-containing transcription factor VRN1 OS=Arabidopsis thaliana GN=VRN1 PE=2 SV=1 |
| Pm004199_0 | MYB | transcription factor | http://planttfdb.cbi.pku.edu.cn/family.php?fam=MYB | MYB3_ARATH Transcription factor MYB3 OS=Arabidopsis thaliana GN=MYB3 PE=1 SV=1 |
| Pm004328_0 | AUX/IAA | transcriptional regulator | -- | IAA8_ARATH Auxin-responsive protein IAA8 OS=Arabidopsis thaliana GN=IAA8 PE=1 SV=1 |
| Pm004406_0 | MYB | transcription factor | http://planttfdb.cbi.pku.edu.cn/family.php?fam=MYB | -- |
| Pm004476_0 | HB | transcription factor | -- | ATB16_ARATH Homeobox-leucine zipper protein ATHB-16 OS=Arabidopsis thaliana GN=ATHB-16 PE=2 SV=2 |
| Pm004718_0 | MADS | transcription factor | -- | MADS2_PETHY Floral homeotic protein PMADS 2 OS=Petunia hybrida GN=PMADS2 PE=2 SV=1 |
| Pm004758_0 | NAC | transcription factor | http://planttfdb.cbi.pku.edu.cn/family.php?fam=NAC | NAC2_ARATH NAC domain-containing protein 2 OS=Arabidopsis thaliana GN=NAC002 PE=2 SV=2 |
| Pm004824_0 | AP2-EREBP | transcription factor | -- | RAP24_ARATH Ethylene-responsive transcription factor RAP2-4 OS=Arabidopsis thaliana GN=RAP2-4 PE=1 SV=1 |
| Pm005087_0 | bZIP | transcription factor | http://planttfdb.cbi.pku.edu.cn/family.php?fam=bZIP | OP2_MAIZE Regulatory protein opaque-2 OS=Zea mays GN=O2 PE=1 SV=1 |
| Pm005161_0 | C2C2-GATA | transcription factor | -- | GAT12_ARATH GATA transcription factor 12 OS=Arabidopsis thaliana GN=GATA12 PE=2 SV=1 |
| Pm005312_0 | AP2-EREBP | transcription factor | -- | RA210_ARATH Ethylene-responsive transcription factor RAP2-10 OS=Arabidopsis thaliana GN=RAP2-10 PE=2 SV=1 |
| Pm005332_0 | MYB | transcription factor | http://planttfdb.cbi.pku.edu.cn/family.php?fam=MYB | MYB44_ARATH Transcription factor MYB44 OS=Arabidopsis thaliana GN=MYB44 PE=2 SV=1 |
| Pm005411_0 | LIM | transcription factor | -- | SF3_HELAN Pollen-specific protein SF3 OS=Helianthus annuus GN=SF3 PE=2 SV=1 |
| Pm005519_0 | HSF | transcription factor | http://planttfdb.cbi.pku.edu.cn/family.php?fam=HSF | HSF30_SOLPE Heat shock factor protein HSF30 OS=Solanum peruvianum GN=HSF30 PE=2 SV=1 |
| Pm005570_0 | MYB | transcription factor | http://planttfdb.cbi.pku.edu.cn/family.php?fam=MYB | MYBC_MAIZE Anthocyanin regulatory C1 protein OS=Zea mays GN=C1 PE=2 SV=1 |
| Pm005577_0 | MYB | transcription factor | http://planttfdb.cbi.pku.edu.cn/family.php?fam=MYB | MYB5_ARATH Transcription repressor MYB5 OS=Arabidopsis thaliana GN=MYB5 PE=1 SV=1 |
| Pm005638_0 | C2C2-CO-like | transcription factor | -- | COL4_ARATH Zinc finger protein CONSTANS-LIKE 4 OS=Arabidopsis thaliana GN=COL4 PE=2 SV=2 |
| Pm005837_0 | TUB | transcription factor | -- | TLP6_ARATH Tubby-like F-box protein 6 OS=Arabidopsis thaliana GN=TULP6 PE=2 SV=1 |
| Pm005877_0 | ARR-B | transcription factor | http://planttfdb.cbi.pku.edu.cn/family.php?fam=ARR-B | ARR2_ARATH Two-component response regulator ARR2 OS=Arabidopsis thaliana GN=ARR2 PE=1 SV=1 |
| Pm005924_0 | BBR/BPC | transcription factor | http://planttfdb.cbi.pku.edu.cn/family.php?fam=BBR-BPC | -- |
| Pm006221_0 | C2H2 | transcription factor | http://planttfdb.cbi.pku.edu.cn/family.php?fam=C2H2 | -- |
| Pm006237_0 | ARF | transcription factor | http://planttfdb.cbi.pku.edu.cn/family.php?fam=ARF | ARFE_ARATH Auxin response factor 5 OS=Arabidopsis thaliana GN=ARF5 PE=1 SV=3 |
| Pm006290_0 | HB | transcription factor | -- | BLH3_ARATH BEL1-like homeodomain protein 3 OS=Arabidopsis thaliana GN=BLH3 PE=1 SV=1 |
| Pm006304_0 | GRAS | transcription factor | http://planttfdb.cbi.pku.edu.cn/family.php?fam=GRAS | GAIPB_CUCMA DELLA protein GAIP-B OS=Cucurbita maxima GN=GAIPB PE=2 SV=1 |
| Pm006324_0 | Tify | transcription factor | -- | TIF6B_ARATH Protein TIFY 6B OS=Arabidopsis thaliana GN=TIFY6B PE=1 SV=1 |
| Pm006354_0 | Orphans | transcriptional regulator | -- | SNL4_ARATH Paired amphipathic helix protein Sin3-like 4 OS=Arabidopsis thaliana GN=SNL4 PE=3 SV=2 |
| Pm006470_0 | NAC | transcription factor | http://planttfdb.cbi.pku.edu.cn/family.php?fam=NAC | NAC29_ARATH NAC domain-containing protein 29 OS=Arabidopsis thaliana GN=NAC029 PE=2 SV=1 |
| Pm006485_0 | C2C2-Dof | transcription factor | -- | DOF52_ARATH Dof zinc finger protein DOF5.2 OS=Arabidopsis thaliana GN=DOF5.2 PE=1 SV=2 |
| Pm006502_0 | zf-HD | transcription factor | http://planttfdb.cbi.pku.edu.cn/family.php?fam=ZF-HD | Y4466_ARATH ZF-HD homeobox protein At4g24660 OS=Arabidopsis thaliana GN=At4g24660 PE=1 SV=1 |
| Pm006530_0 | HB | transcription factor | -- | ATB13_ARATH Homeobox-leucine zipper protein ATHB-13 OS=Arabidopsis thaliana GN=ATHB-13 PE=2 SV=2 |
| Pm006609_0 | bHLH | transcription factor | http://planttfdb.cbi.pku.edu.cn/family.php?fam=bHLH | BPE_ARATH Transcription factor BPE OS=Arabidopsis thaliana GN=BPE PE=2 SV=1 |
| Pm007027_0 | Orphans | transcriptional regulator | -- | AHK3_ARATH Histidine kinase 3 OS=Arabidopsis thaliana GN=AHK3 PE=1 SV=1 |
| Pm007035_0 | SBP | transcription factor | http://planttfdb.cbi.pku.edu.cn/family.php?fam=SBP | SPL13_ARATH Squamosa promoter-binding-like protein 13 OS=Arabidopsis thaliana GN=SPL13 PE=2 SV=2 |
| Pm007070_0 | NAC | transcription factor | http://planttfdb.cbi.pku.edu.cn/family.php?fam=NAC | NAC68_ORYSJ NAC domain-containing protein 68 OS=Oryza sativa subsp. japonica GN=NAC68 PE=2 SV=1 |
| Pm007084_0 | AP2-EREBP | transcription factor | -- | EF113_ARATH Ethylene-responsive transcription factor ERF113 OS=Arabidopsis thaliana GN=ERF113 PE=2 SV=1 |
| Pm007163_0 | bZIP | transcription factor | http://planttfdb.cbi.pku.edu.cn/family.php?fam=bZIP | HYH_ARATH Transcription factor HY5-like OS=Arabidopsis thaliana GN=HYH PE=1 SV=1 |
| Pm007178_0 | Orphans | transcriptional regulator | -- | COL14_ARATH Zinc finger protein CONSTANS-LIKE 14 OS=Arabidopsis thaliana GN=COL14 PE=2 SV=2 |
| Pm007529_0 | Orphans | transcriptional regulator | -- | STO_ARATH Salt tolerance protein OS=Arabidopsis thaliana GN=STO PE=1 SV=1 |
| Pm008000_0 | CCAAT | transcription factor | -- | NFYC9_ARATH Nuclear transcription factor Y subunit C-9 OS=Arabidopsis thaliana GN=NFYC9 PE=2 SV=1 |
| Pm008113_0 | TUB | transcription factor | -- | TLP3_ARATH Tubby-like F-box protein 3 OS=Arabidopsis thaliana GN=TULP3 PE=2 SV=1 |
| Pm008146_0 | C3H | transcription factor | http://planttfdb.cbi.pku.edu.cn/family.php?fam=C3H | C3H58_ARATH Zinc finger CCCH domain-containing protein 58 OS=Arabidopsis thaliana GN=At5g18550 PE=2 SV=1 |
| Pm008283_0 | ARR-B | transcription factor | http://planttfdb.cbi.pku.edu.cn/family.php?fam=ARR-B | ARR1_ARATH Two-component response regulator ARR1 OS=Arabidopsis thaliana GN=ARR1 PE=1 SV=2 |
| Pm008425_0 | MYB | transcription factor | http://planttfdb.cbi.pku.edu.cn/family.php?fam=MYB | MYB05_ANTMA Myb-related protein 305 OS=Antirrhinum majus GN=MYB305 PE=2 SV=1 |
| Pm008522_0 | C2C2-CO-like | transcription factor | -- | COL5_ARATH Zinc finger protein CONSTANS-LIKE 5 OS=Arabidopsis thaliana GN=COL5 PE=2 SV=2 |
| Pm008629_0 | AP2-EREBP | transcription factor | -- | AP2L1_ARATH AP2-like ethylene-responsive transcription factor At1g16060 OS=Arabidopsis thaliana GN=At1g16060 PE=2 SV=1 |
| Pm008898_0 | bHLH | transcription factor | http://planttfdb.cbi.pku.edu.cn/family.php?fam=bHLH | BH035_ARATH Transcription factor bHLH35 OS=Arabidopsis thaliana GN=BHLH35 PE=2 SV=1 |
| Pm008963_0 | ARF | transcription factor | http://planttfdb.cbi.pku.edu.cn/family.php?fam=ARF | ARFS_ORYSJ Auxin response factor 19 OS=Oryza sativa subsp. japonica GN=ARF19 PE=2 SV=2 |
| Pm009169_0 | AUX/IAA | transcriptional regulator | -- | IAA14_ARATH Auxin-responsive protein IAA14 OS=Arabidopsis thaliana GN=IAA14 PE=1 SV=2 |
| Pm009170_0 | AUX/IAA | transcriptional regulator | -- | IAA4_PEA Auxin-induced protein IAA4 OS=Pisum sativum GN=IAA4/5 PE=1 SV=1 |
| Pm009237_0 | HSF | transcription factor | http://planttfdb.cbi.pku.edu.cn/family.php?fam=HSF | HFA6B_ARATH Heat stress transcription factor A-6b OS=Arabidopsis thaliana GN=HSFA6b PE=2 SV=1 |
| Pm009413_0 | SNF2 | transcriptional regulator | -- | SM3L3_ARATH Putative SWI/SNF-related matrix-associated actin-dependent regulator of chromatin subfamily A member 3-like 3 OS=Arabidopsis thaliana GN=At5g43530 PE=3 SV=1 |
| Pm009418_0 | Orphans | transcriptional regulator | -- | ETR2_ARATH Ethylene receptor 2 OS=Arabidopsis thaliana GN=ETR2 PE=1 SV=2 |
| Pm009679_0 | WRKY | transcription factor | http://planttfdb.cbi.pku.edu.cn/family.php?fam=WRKY | WRKY6_ARATH WRKY transcription factor 6 OS=Arabidopsis thaliana GN=WRKY6 PE=1 SV=1 |
| Pm009748_0 | MYB | transcription factor | http://planttfdb.cbi.pku.edu.cn/family.php?fam=MYB | CPC_ARATH Transcription factor CPC OS=Arabidopsis thaliana GN=CPC PE=1 SV=1 |
| Pm009955_0 | NAC | transcription factor | http://planttfdb.cbi.pku.edu.cn/family.php?fam=NAC | NAC18_ARATH NAC domain-containing protein 18 OS=Arabidopsis thaliana GN=NAC018 PE=2 SV=1 |
| Pm009975_0 | C2C2-Dof | transcription factor | -- | DOF53_ARATH Dof zinc finger protein DOF5.3 OS=Arabidopsis thaliana GN=DOF5.3 PE=2 SV=1 |
| Pm010075_0 | SBP | transcription factor | http://planttfdb.cbi.pku.edu.cn/family.php?fam=SBP | SBP1_ANTMA Squamosa promoter-binding protein 1 OS=Antirrhinum majus GN=SBP1 PE=2 SV=1 |
| Pm010083_0 | AP2-EREBP | transcription factor | -- | ERF81_ARATH Ethylene-responsive transcription factor 12 OS=Arabidopsis thaliana GN=ERF12 PE=2 SV=1 |
| Pm010126_0 | C3H | transcription factor | http://planttfdb.cbi.pku.edu.cn/family.php?fam=C3H | GDL84_ARATH GDSL esterase/lipase At5g45920 OS=Arabidopsis thaliana GN=At5g45920 PE=2 SV=1 |
| Pm010170_0 | BES1 | transcription factor | http://planttfdb.cbi.pku.edu.cn/family.php?fam=BES1 | BEH4_ARATH BES1/BZR1 homolog protein 4 OS=Arabidopsis thaliana GN=BEH4 PE=1 SV=1 |
| Pm010346_0 | MADS | transcription factor | -- | AG_PANGI Floral homeotic protein AGAMOUS OS=Panax ginseng GN=AG2 PE=2 SV=1 |
| Pm010368_0 | AP2-EREBP | transcription factor | -- | PTI5_SOLLC Pathogenesis-related genes transcriptional activator PTI5 OS=Solanum lycopersicum GN=PTI5 PE=2 SV=1 |
| Pm010440_0 | TCP | transcription factor | http://planttfdb.cbi.pku.edu.cn/family.php?fam=TCP | TCP2_ARATH Transcription factor TCP2 OS=Arabidopsis thaliana GN=TCP2 PE=2 SV=1 |
| Pm010628_0 | Jumonji | transcriptional regulator | -- | KDM5A_MOUSE Lysine-specific demethylase 5A OS=Mus musculus GN=Kdm5a PE=1 SV=2 |
| Pm011249_0 | Orphans | transcriptional regulator | -- | STHY_ARATH Probable salt tolerance-like protein At1g78600 OS=Arabidopsis thaliana GN=At1g78600 PE=2 SV=2 |
| Pm011353_0 | bZIP | transcription factor | http://planttfdb.cbi.pku.edu.cn/family.php?fam=bZIP | VIP1_ARATH Transcription factor VIP1 OS=Arabidopsis thaliana GN=VIP1 PE=1 SV=1 |
| Pm011369_0 | TCP | transcription factor | http://planttfdb.cbi.pku.edu.cn/family.php?fam=TCP | TCP4_ARATH Transcription factor TCP4 OS=Arabidopsis thaliana GN=TCP4 PE=2 SV=1 |
| Pm011391_0 | AP2-EREBP | transcription factor | -- | ERF78_ARATH Ethylene-responsive transcription factor 4 OS=Arabidopsis thaliana GN=ERF4 PE=1 SV=1 |
| Pm011603_0 | NAC | transcription factor | http://planttfdb.cbi.pku.edu.cn/family.php?fam=NAC | NAC72_ARATH NAC domain-containing protein 72 OS=Arabidopsis thaliana GN=NAC072 PE=2 SV=1 |
| Pm011604_0 | NAC | transcription factor | http://planttfdb.cbi.pku.edu.cn/family.php?fam=NAC | NAC18_ARATH NAC domain-containing protein 18 OS=Arabidopsis thaliana GN=NAC018 PE=2 SV=1 |
| Pm012019_0 | GRAS | transcription factor | http://planttfdb.cbi.pku.edu.cn/family.php?fam=GRAS | PAT1_ARATH Scarecrow-like transcription factor PAT1 OS=Arabidopsis thaliana GN=PAT1 PE=2 SV=1 |
| Pm012309_0 | IWS1 | transcriptional regulator | -- | -- |
| Pm012630_0 | NAC | transcription factor | http://planttfdb.cbi.pku.edu.cn/family.php?fam=NAC | NAC2_ARATH NAC domain-containing protein 2 OS=Arabidopsis thaliana GN=NAC002 PE=2 SV=2 |
| Pm012968_0 | ARF | transcription factor | http://planttfdb.cbi.pku.edu.cn/family.php?fam=ARF | ARFH_ARATH Auxin response factor 8 OS=Arabidopsis thaliana GN=ARF8 PE=2 SV=2 |
| Pm012998_0 | MYB | transcription factor | http://planttfdb.cbi.pku.edu.cn/family.php?fam=MYB | MYBG_DICDI Myb-like protein G OS=Dictyosteliumdiscoideum GN=mybG PE=3 SV=1 |
| Pm013051_0 | Orphans | transcriptional regulator | -- | COL2_ARATH Zinc finger protein CONSTANS-LIKE 2 OS=Arabidopsis thaliana GN=COL2 PE=1 SV=1 |
| Pm013132_0 | G2-like | transcription factor | http://planttfdb.cbi.pku.edu.cn/family.php?fam=G2-like | APL_ARATH Myb family transcription factor APL OS=Arabidopsis thaliana GN=APL PE=2 SV=2 |
| Pm013141_0 | HB | transcription factor | -- | -- |
| Pm013228_0 | Tify | transcription factor | -- | TI10B_ARATH Protein TIFY 10B OS=Arabidopsis thaliana GN=TIFY10B PE=1 SV=1 |
| Pm013277_0 | MYB | transcription factor | http://planttfdb.cbi.pku.edu.cn/family.php?fam=MYB | MYB6_ARATH Transcription repressor MYB6 OS=Arabidopsis thaliana GN=MYB6 PE=1 SV=1 |
| Pm013279_0 | MYB | transcription factor | http://planttfdb.cbi.pku.edu.cn/family.php?fam=MYB | TT2_ARATH Transcription factor TT2 OS=Arabidopsis thaliana GN=TT2 PE=1 SV=1 |
| Pm013373_0 | C2C2-Dof | transcription factor | -- | DOF17_ARATH Dof zinc finger protein DOF1.7 OS=Arabidopsis thaliana GN=DOF1.7 PE=2 SV=1 |
| Pm013416_0 | AUX/IAA | transcriptional regulator | -- | IAA26_ARATH Auxin-responsive protein IAA26 OS=Arabidopsis thaliana GN=IAA26 PE=1 SV=2 |
| Pm013440_0 | C2C2-GATA | transcription factor | -- | GATA9_ARATH GATA transcription factor 9 OS=Arabidopsis thaliana GN=GATA9 PE=2 SV=1 |
| Pm013549_0 | HB | transcription factor | -- | HGL2_ARATH Homeobox-leucine zipper protein GLABRA 2 OS=Arabidopsis thaliana GN=GL2 PE=2 SV=3 |
| Pm013596_0 | AUX/IAA | transcriptional regulator | -- | AUX22_SOYBN Auxin-induced protein AUX22 OS=Glycine max GN=AUX22 PE=2 SV=1 |
| Pm013597_0 | AUX/IAA | transcriptional regulator | -- | IAA17_ARATH Auxin-responsive protein IAA17 OS=Arabidopsis thaliana GN=IAA17 PE=1 SV=2 |
| Pm013856_0 | AP2-EREBP | transcription factor | -- | AP2L1_ARATH AP2-like ethylene-responsive transcription factor At1g16060 OS=Arabidopsis thaliana GN=At1g16060 PE=2 SV=1 |
| Pm014146_0 | SNF2 | transcriptional regulator | -- | RAD26_MOUSE Putative DNA repair and recombination protein RAD26-like OS=Mus musculus GN=Rad26l PE=2 SV=2 |
| Pm014253_0 | bZIP | transcription factor | http://planttfdb.cbi.pku.edu.cn/family.php?fam=bZIP | RF2B_ORYSJ Transcription factor RF2b OS=Oryza sativa subsp. japonica GN=RF2b PE=1 SV=1 |
| Pm014372_0 | MYB | transcription factor | http://planttfdb.cbi.pku.edu.cn/family.php?fam=MYB | MYB08_ANTMA Myb-related protein 308 OS=Antirrhinum majus GN=MYB308 PE=2 SV=1 |
| Pm014733_0 | ARR-B | transcription factor | http://planttfdb.cbi.pku.edu.cn/family.php?fam=ARR-B | APRR2_ARATH Two-component response regulator-like APRR2 OS=Arabidopsis thaliana GN=APRR2 PE=2 SV=2 |
| Pm014818_0 | ARF | transcription factor | http://planttfdb.cbi.pku.edu.cn/family.php?fam=ARF | ARFF_ARATH Auxin response factor 6 OS=Arabidopsis thaliana GN=ARF6 PE=2 SV=2 |
| Pm014937_0 | C2C2-CO-like | transcription factor | -- | COL9_ARATH Zinc finger protein CONSTANS-LIKE 9 OS=Arabidopsis thaliana GN=COL9 PE=2 SV=1 |
| Pm015020_0 | WRKY | transcription factor | http://planttfdb.cbi.pku.edu.cn/family.php?fam=WRKY | WRKY1_ARATH WRKY transcription factor 1 OS=Arabidopsis thaliana GN=WRKY1 PE=1 SV=1 |
| Pm015234_0 | Orphans | transcriptional regulator | -- | COL14_ARATH Zinc finger protein CONSTANS-LIKE 14 OS=Arabidopsis thaliana GN=COL14 PE=2 SV=2 |
| Pm015294_0 | Orphans | transcriptional regulator | -- | COL11_ARATH Putative zinc finger protein CONSTANS-LIKE 11 OS=Arabidopsis thaliana GN=COL11 PE=2 SV=2 |
| Pm015446_0 | C2C2-CO-like | transcription factor | -- | COL2_ARATH Zinc finger protein CONSTANS-LIKE 2 OS=Arabidopsis thaliana GN=COL2 PE=1 SV=1 |
| Pm015500_0 | AP2-EREBP | transcription factor | -- | RAV2_ARATH AP2/ERF and B3 domain-containing transcription repressor RAV2 OS=Arabidopsis thaliana GN=RAV2 PE=2 SV=1 |
| Pm015540_1 | LOB | transcription factor | -- | LBD41_ARATH LOB domain-containing protein 41 OS=Arabidopsis thaliana GN=LBD41 PE=2 SV=1 |
| Pm015758_0 | IWS1 | transcriptional regulator | -- | -- |
| Pm015802_0 | Orphans | transcriptional regulator | -- | AB15B_ARATH ABC transporter B family member 15 OS=Arabidopsis thaliana GN=ABCB15 PE=1 SV=1 |
| Pm015880_0 | MYB | transcription factor | http://planttfdb.cbi.pku.edu.cn/family.php?fam=MYB | MYB06_ANTMA Myb-related protein 306 OS=Antirrhinum majus GN=MYB306 PE=2 SV=1 |
| Pm015896_0 | C2C2-Dof | transcription factor | -- | DOF52_ARATH Dof zinc finger protein DOF5.2 OS=Arabidopsis thaliana GN=DOF5.2 PE=1 SV=2 |
| Pm015914_0 | NAC | transcription factor | http://planttfdb.cbi.pku.edu.cn/family.php?fam=NAC | NC100_ARATH NAC domain-containing protein 100 OS=Arabidopsis thaliana GN=NAC100 PE=2 SV=1 |
| Pm015937_0 | WRKY | transcription factor | http://planttfdb.cbi.pku.edu.cn/family.php?fam=WRKY | WRKY4_ARATH Probable WRKY transcription factor 4 OS=Arabidopsis thaliana GN=WRKY4 PE=1 SV=2 |
| Pm016049_0 | TCP | transcription factor | http://planttfdb.cbi.pku.edu.cn/family.php?fam=TCP | TCP13_ARATH Transcription factor TCP13 OS=Arabidopsis thaliana GN=TCP13 PE=2 SV=1 |
| Pm016092_4 | ARF | transcription factor | http://planttfdb.cbi.pku.edu.cn/family.php?fam=ARF | AK3_ARATH Aspartokinase 3, chloroplastic OS=Arabidopsis thaliana GN=AK3 PE=1 SV=1 |
| Pm016093_0 | bHLH | transcription factor | http://planttfdb.cbi.pku.edu.cn/family.php?fam=bHLH | ICE1_ARATH Transcription factor ICE1 OS=Arabidopsis thaliana GN=SCRM PE=1 SV=1 |
| Pm016138_0 | SBP | transcription factor | http://planttfdb.cbi.pku.edu.cn/family.php?fam=SBP | SPL13_ARATH Squamosa promoter-binding-like protein 13 OS=Arabidopsis thaliana GN=SPL13 PE=2 SV=2 |
| Pm017050_0 | MYB | transcription factor | http://planttfdb.cbi.pku.edu.cn/family.php?fam=MYB | MY1R1_SOLTU Transcription factor MYB1R1 OS=Solanum tuberosum PE=2 SV=1 |
| Pm017547_0 | NAC | transcription factor | http://planttfdb.cbi.pku.edu.cn/family.php?fam=NAC | NAC78_ARATH NAC domain-containing protein 78 OS=Arabidopsis thaliana GN=NAC078 PE=2 SV=2 |
| Pm017609_0 | Jumonji | transcriptional regulator | -- | KDM3B_HUMAN Lysine-specific demethylase 3B OS=Homo sapiens GN=KDM3B PE=1 SV=2 |
| Pm017800_0 | bHLH | transcription factor | http://planttfdb.cbi.pku.edu.cn/family.php?fam=bHLH | UNE10_ARATH Transcription factor UNE10 OS=Arabidopsis thaliana GN=UNE10 PE=2 SV=1 |
| Pm017821_0 | GRAS | transcription factor | http://planttfdb.cbi.pku.edu.cn/family.php?fam=GRAS | SCL6_ARATH Scarecrow-like protein 6 OS=Arabidopsis thaliana GN=SCL6 PE=1 SV=1 |
| Pm017936_0 | LOB | transcription factor | -- | LBD18_ARATH LOB domain-containing protein 18 OS=Arabidopsis thaliana GN=LBD18 PE=2 SV=2 |
| Pm017987_0 | HB | transcription factor | -- | ANL2_ARATH Homeobox-leucine zipper protein ANTHOCYANINLESS 2 OS=Arabidopsis thaliana GN=ANL2 PE=2 SV=1 |
| Pm018055_0 | LIM | transcription factor | -- | SF3_HELAN Pollen-specific protein SF3 OS=Helianthus annuus GN=SF3 PE=2 SV=1 |
| Pm018100_0 | TCP | transcription factor | http://planttfdb.cbi.pku.edu.cn/family.php?fam=TCP | TCP9_ARATH Transcription factor TCP9 OS=Arabidopsis thaliana GN=TCP9 PE=2 SV=1 |
| Pm018108_0 | C3H | transcription factor | http://planttfdb.cbi.pku.edu.cn/family.php?fam=C3H | C3H48_ARATH Zinc finger CCCH domain-containing protein 48 OS=Arabidopsis thaliana GN=ZFWD1 PE=2 SV=1 |
| Pm018226_0 | MYB | transcription factor | http://planttfdb.cbi.pku.edu.cn/family.php?fam=MYB | ASG4_ARATH Transcription factor ASG4 OS=Arabidopsis thaliana GN=ASG4 PE=2 SV=1 |
| Pm018314_0 | HB | transcription factor | -- | ATHB7_ARATH Homeobox-leucine zipper protein ATHB-7 OS=Arabidopsis thaliana GN=ATHB-7 PE=2 SV=2 |
| Pm018355_0 | bHLH | transcription factor | http://planttfdb.cbi.pku.edu.cn/family.php?fam=bHLH | BH013_ARATH Transcription factor bHLH13 OS=Arabidopsis thaliana GN=BHLH13 PE=2 SV=1 |
| Pm018359_0 | Jumonji | transcriptional regulator | -- | CNX3_ARATH Molybdopterin biosynthesis protein CNX3 OS=Arabidopsis thaliana GN=CNX3 PE=2 SV=1 |
| Pm018422_0 | bZIP | transcription factor | http://planttfdb.cbi.pku.edu.cn/family.php?fam=bZIP | CPRF1_PETCR Common plant regulatory factor 1 OS=Petroselinum crispum GN=CPRF1 PE=2 SV=1 |
| Pm018532_0 | C2C2-GATA | transcription factor | -- | GATA8_ARATH GATA transcription factor 8 OS=Arabidopsis thaliana GN=GATA8 PE=2 SV=1 |
| Pm018533_0 | ARF | transcription factor | http://planttfdb.cbi.pku.edu.cn/family.php?fam=ARF | ARFR_ORYSJ Auxin response factor 18 OS=Oryza sativa subsp. japonica GN=ARF18 PE=2 SV=1 |
| Pm018608_0 | AP2-EREBP | transcription factor | -- | RAP27_ARATH Ethylene-responsive transcription factor RAP2-7 OS=Arabidopsis thaliana GN=RAP2-7 PE=2 SV=2 |
| Pm018761_0 | C2H2 | transcription factor | http://planttfdb.cbi.pku.edu.cn/family.php?fam=C2H2 | ZFP1_WHEAT Zinc finger protein 1 OS=Triticum aestivum PE=2 SV=1 |
| Pm018870_0 | LIM | transcription factor | -- | SF3_HELAN Pollen-specific protein SF3 OS=Helianthus annuus GN=SF3 PE=2 SV=1 |
| Pm018913_0 | HMG | transcriptional regulator | -- | HMGB1_ARATH High mobility group B protein 1 OS=Arabidopsis thaliana GN=HMGB1 PE=1 SV=1 |
| Pm018988_0 | SNF2 | transcriptional regulator | -- | RAD54_DROAN DNA repair and recombination protein RAD54-like OS=Drosophila ananassae GN=okr PE=3 SV=1 |
| Pm018989_0 | AP2-EREBP | transcription factor | -- | DRE2C_ARATH Dehydration-responsive element-binding protein 2C OS=Arabidopsis thaliana GN=DREB2C PE=2 SV=2 |
| Pm019060_0 | bZIP | transcription factor | http://planttfdb.cbi.pku.edu.cn/family.php?fam=bZIP | RF2B_ORYSJ Transcription factor RF2b OS=Oryza sativa subsp. japonica GN=RF2b PE=1 SV=1 |
| Pm019118_0 | PHD | transcriptional regulator | -- | JADE1_DANRE Protein Jade-1 OS=Danio rerio GN=phf17 PE=2 SV=1 |
| Pm019386_0 | AP2-EREBP | transcription factor | -- | DRE1D_ARATH Dehydration-responsive element-binding protein 1D OS=Arabidopsis thaliana GN=DREB1D PE=2 SV=1 |
| Pm019545_0 | AP2-EREBP | transcription factor | -- | ERF5_TOBAC Ethylene-responsive transcription factor 5 OS=Nicotiana tabacum GN=ERF5 PE=2 SV=1 |
| Pm019659_0 | NAC | transcription factor | http://planttfdb.cbi.pku.edu.cn/family.php?fam=NAC | NAC78_ARATH NAC domain-containing protein 78 OS=Arabidopsis thaliana GN=NAC078 PE=2 SV=2 |
| Pm019675_0 | Pseudo ARR-B | transcriptional regulator | -- | PRR95_ORYSJ Two-component response regulator-like PRR95 OS=Oryza sativa subsp. japonica GN=PRR95 PE=2 SV=1 |
| Pm019687_0 | GRF | transcription factor | http://planttfdb.cbi.pku.edu.cn/family.php?fam=GRF | -- |
| Pm019693_0 | HB | transcription factor | -- | ATHB7_ARATH Homeobox-leucine zipper protein ATHB-7 OS=Arabidopsis thaliana GN=ATHB-7 PE=2 SV=2 |
| Pm019710_0 | C2C2-Dof | transcription factor | -- | DOF46_ARATH Dof zinc finger protein DOF4.6 OS=Arabidopsis thaliana GN=DOF4.6 PE=2 SV=2 |
| Pm019745_0 | GNAT | transcriptional regulator | -- | -- |
| Pm019782_0 | Sigma70-like | transcription factor | -- | RPOD_NOSS1 RNA polymerase sigma factor rpoD OS=Nostoc sp. (strain PCC 7120 / UTEX 2576) GN=rpoD PE=3 SV=1 |
| Pm019863_0 | GRAS | transcription factor | http://planttfdb.cbi.pku.edu.cn/family.php?fam=GRAS | SCL8_ARATH Scarecrow-like protein 8 OS=Arabidopsis thaliana GN=SCL8 PE=2 SV=1 |
| Pm019885_0 | BSD | transcription factor | -- | BSDC1_DANRE BSD domain-containing protein 1 OS=Danio rerio GN=bsdc1 PE=1 SV=1 |
| Pm019959_0 | C2C2-CO-like | transcription factor | -- | COL13_ARATH Zinc finger protein CONSTANS-LIKE 13 OS=Arabidopsis thaliana GN=COL13 PE=2 SV=1 |
| Pm020059_0 | AP2-EREBP | transcription factor | -- | RAP23_ARATH Ethylene-responsive transcription factor RAP2-3 OS=Arabidopsis thaliana GN=RAP2-3 PE=1 SV=2 |
| Pm020069_0 | WRKY | transcription factor | http://planttfdb.cbi.pku.edu.cn/family.php?fam=WRKY | WRK35_ARATH Probable WRKY transcription factor 35 OS=Arabidopsis thaliana GN=WRKY35 PE=2 SV=1 |
| Pm020070_0 | GRAS | transcription factor | http://planttfdb.cbi.pku.edu.cn/family.php?fam=GRAS | GAI1_VITVI DELLA protein GAI1 OS=Vitis vinifera GN=GAI1 PE=2 SV=1 |
| Pm020071_0 | SET | transcriptional regulator | -- | EZA1_ARATH Histone-lysine N-methyltransferase EZA1 OS=Arabidopsis thaliana GN=EZA1 PE=2 SV=1 |
| Pm020080_0 | bZIP | transcription factor | http://planttfdb.cbi.pku.edu.cn/family.php?fam=bZIP | OCS1_MAIZE Ocs element-binding factor 1 OS=Zea mays GN=OBF1 PE=2 SV=2 |
| Pm020227_0 | AUX/IAA | transcriptional regulator | -- | AX22D_VIGRR Auxin-induced protein 22D OS=Vigna radiata var. radiata GN=AUX22D PE=2 SV=1 |
| Pm020229_0 | bHLH | transcription factor | http://planttfdb.cbi.pku.edu.cn/family.php?fam=bHLH | UNE12_ARATH Transcription factor UNE12 OS=Arabidopsis thaliana GN=UNE12 PE=2 SV=2 |
| Pm020363_0 | LUG | transcriptional regulator | -- | LEUNG_ARATH Transcriptional corepressor LEUNIG OS=Arabidopsis thaliana GN=LUG PE=1 SV=2 |
| Pm020501_0 | AUX/IAA | transcriptional regulator | -- | IAA11_ARATH Auxin-responsive protein IAA11 OS=Arabidopsis thaliana GN=IAA11 PE=1 SV=1 |
| Pm020580_0 | NAC | transcription factor | http://planttfdb.cbi.pku.edu.cn/family.php?fam=NAC | NAC22_ARATH NAC domain-containing protein 21/22 OS=Arabidopsis thaliana GN=NAC021 PE=1 SV=2 |
| Pm020721_2 | C2H2 | transcription factor | http://planttfdb.cbi.pku.edu.cn/family.php?fam=C2H2 | UGAL2_ARATH UDP-galactose transporter 2 OS=Arabidopsis thaliana GN=UDP-GALT2 PE=2 SV=1 |
| Pm021139_0 | MYB | transcription factor | http://planttfdb.cbi.pku.edu.cn/family.php?fam=MYB | H1_MAIZE Histone H1 OS=Zea mays PE=2 SV=2 |
| Pm021141_0 | LIM | transcription factor | -- | SF3_HELAN Pollen-specific protein SF3 OS=Helianthus annuus GN=SF3 PE=2 SV=1 |
| Pm021211_0 | MYB | transcription factor | http://planttfdb.cbi.pku.edu.cn/family.php?fam=MYB | MYB44_ARATH Transcription factor MYB44 OS=Arabidopsis thaliana GN=MYB44 PE=2 SV=1 |
| Pm021321_0 | AP2-EREBP | transcription factor | -- | EF109_ARATH Ethylene-responsive transcription factor ERF109 OS=Arabidopsis thaliana GN=ERF109 PE=2 SV=1 |
| Pm021430_0 | bZIP | transcription factor | http://planttfdb.cbi.pku.edu.cn/family.php?fam=bZIP | AI5L6_ARATH ABSCISIC ACID-INSENSITIVE 5-like protein 6 OS=Arabidopsis thaliana GN=ABF3 PE=1 SV=1 |
| Pm021642_0 | bZIP | transcription factor | http://planttfdb.cbi.pku.edu.cn/family.php?fam=bZIP | -- |
| Pm021662_0 | C3H | transcription factor | http://planttfdb.cbi.pku.edu.cn/family.php?fam=C3H | DUS3L_ARATH tRNA-dihydrouridine synthase 3-like OS=Arabidopsis thaliana GN=At4g38890 PE=2 SV=1 |
| Pm021705_0 | C2C2-GATA | transcription factor | -- | GATA5_ARATH GATA transcription factor 5 OS=Arabidopsis thaliana GN=GATA5 PE=2 SV=1 |
| Pm021762_0 | MYB | transcription factor | http://planttfdb.cbi.pku.edu.cn/family.php?fam=MYB | -- |
| Pm021823_0 | HB | transcription factor | -- | BLH6_ARATH BEL1-like homeodomain protein 6 OS=Arabidopsis thaliana GN=BLH6 PE=2 SV=1 |
| Pm021827_0 | PHD | transcriptional regulator | -- | PHF2_HUMAN PHD finger protein 2 OS=Homo sapiens GN=PHF2 PE=1 SV=4 |
| Pm021850_0 | Orphans | transcriptional regulator | -- | STHX_ARATH Probable salt tolerance-like protein At1g75540 OS=Arabidopsis thaliana GN=At1g75540 PE=2 SV=1 |
| Pm022081_0 | C2H2 | transcription factor | http://planttfdb.cbi.pku.edu.cn/family.php?fam=C2H2 | MBD8_ARATH Methyl-CpG-binding domain-containing protein 8 OS=Arabidopsis thaliana GN=MBD8 PE=2 SV=1 |
| Pm022509_0 | AP2-EREBP | transcription factor | -- | EF114_ARATH Ethylene-responsive transcription factor ERF114 OS=Arabidopsis thaliana GN=ERF114 PE=2 SV=1 |
| Pm022643_0 | C2C2-Dof | transcription factor | -- | DOF12_ARATH Dof zinc finger protein DOF1.2 OS=Arabidopsis thaliana GN=DOF1.2 PE=2 SV=1 |
| Pm022704_0 | GRAS | transcription factor | http://planttfdb.cbi.pku.edu.cn/family.php?fam=GRAS | SCL1_ARATH Scarecrow-like protein 1 OS=Arabidopsis thaliana GN=SCL1 PE=2 SV=1 |
| Pm022874_0 | PLATZ | transcription factor | -- | -- |
| Pm022964_0 | RWP-RK | transcription factor | -- | NLP4_ARATH Protein NLP4 OS=Arabidopsis thaliana GN=NLP4 PE=2 SV=1 |
| Pm023160_0 | Jumonji | transcriptional regulator | -- | KDM5B_MOUSE Lysine-specific demethylase 5B OS=Mus musculus GN=Kdm5b PE=1 SV=1 |
| Pm023207_0 | bHLH | transcription factor | http://planttfdb.cbi.pku.edu.cn/family.php?fam=bHLH | BH096_ARATH Transcription factor bHLH96 OS=Arabidopsis thaliana GN=BHLH96 PE=2 SV=1 |
| Pm023220_0 | PLATZ | transcription factor | -- | -- |
| Pm023419_0 | MYB | transcription factor | http://planttfdb.cbi.pku.edu.cn/family.php?fam=MYB | MYBC_MAIZE Anthocyanin regulatory C1 protein OS=Zea mays GN=C1 PE=2 SV=1 |
| Pm023533_0 | WRKY | transcription factor | http://planttfdb.cbi.pku.edu.cn/family.php?fam=WRKY | WRK21_ARATH Probable WRKY transcription factor 21 OS=Arabidopsis thaliana GN=WRKY21 PE=1 SV=1 |
| Pm023748_0 | TCP | transcription factor | http://planttfdb.cbi.pku.edu.cn/family.php?fam=TCP | TCP9_ARATH Transcription factor TCP9 OS=Arabidopsis thaliana GN=TCP9 PE=2 SV=1 |
| Pm023766_0 | AP2-EREBP | transcription factor | -- | ERF25_ARATH Ethylene-responsive transcription factor ERF025 OS=Arabidopsis thaliana GN=ERF025 PE=2 SV=1 |
| Pm023867_0 | LIM | transcription factor | -- | SF3_HELAN Pollen-specific protein SF3 OS=Helianthus annuus GN=SF3 PE=2 SV=1 |
| Pm024053_0 | AP2-EREBP | transcription factor | -- | ERF61_ARATH Ethylene-responsive transcription factor ERF061 OS=Arabidopsis thaliana GN=ERF061 PE=2 SV=1 |
| Pm024149_0 | ARF | transcription factor | http://planttfdb.cbi.pku.edu.cn/family.php?fam=ARF | ARFI_ARATH Auxin response factor 9 OS=Arabidopsis thaliana GN=ARF9 PE=2 SV=1 |
| Pm024289_0 | Trihelix | transcription factor | http://planttfdb.cbi.pku.edu.cn/family.php?fam=Trihelix | TGT2_ARATH Trihelix transcription factor GT-2 OS=Arabidopsis thaliana GN=GT-2 PE=2 SV=1 |
| Pm024294_0 | FHA | transcription factor | -- | TYDP1_RAT Tyrosyl-DNA phosphodiesterase 1 OS=Rattus norvegicus GN=Tdp1 PE=2 SV=1 |
| Pm024405_0 | TAZ | transcription factor | -- | BT2_ARATH BTB/POZ and TAZ domain-containing protein 2 OS=Arabidopsis thaliana GN=BT2 PE=1 SV=1 |
| Pm024430_0 | C3H | transcription factor | http://planttfdb.cbi.pku.edu.cn/family.php?fam=C3H | C3H67_ARATH Zinc finger CCCH domain-containing protein 67 OS=Arabidopsis thaliana GN=At5g63260 PE=2 SV=2 |
| Pm024446_0 | GNAT | transcriptional regulator | -- | ELP3_ARATH Elongator complex protein 3 OS=Arabidopsis thaliana GN=HAG3 PE=1 SV=1 |
| Pm024491_0 | FHA | transcription factor | -- | ATAD1_RAT ATPase family AAA domain-containing protein 1 OS=Rattus norvegicus GN=Atad1 PE=2 SV=1 |
| Pm024587_0 | bHLH | transcription factor | http://planttfdb.cbi.pku.edu.cn/family.php?fam=bHLH | ICE1_ARATH Transcription factor ICE1 OS=Arabidopsis thaliana GN=SCRM PE=1 SV=1 |
| Pm024588_0 | ARF | transcription factor | http://planttfdb.cbi.pku.edu.cn/family.php?fam=ARF | ARFB_ARATH Auxin response factor 2 OS=Arabidopsis thaliana GN=ARF2 PE=1 SV=2 |
| Pm024636_0 | bHLH | transcription factor | http://planttfdb.cbi.pku.edu.cn/family.php?fam=bHLH | BH079_ARATH Transcription factor bHLH79 OS=Arabidopsis thaliana GN=BHLH79 PE=2 SV=1 |
| Pm024686_0 | LIM | transcription factor | -- | SF3_HELAN Pollen-specific protein SF3 OS=Helianthus annuus GN=SF3 PE=2 SV=1 |
| Pm024690_0 | IWS1 | transcriptional regulator | -- | -- |
| Pm024816_0 | HB | transcription factor | -- | HAT5_ARATH Homeobox-leucine zipper protein HAT5 OS=Arabidopsis thaliana GN=HAT5 PE=1 SV=1 |
| Pm024862_0 | Pseudo ARR-B | transcriptional regulator | -- | APRR1_ARATH Two-component response regulator-like APRR1 OS=Arabidopsis thaliana GN=APRR1 PE=1 SV=1 |
| Pm024887_0 | NAC | transcription factor | http://planttfdb.cbi.pku.edu.cn/family.php?fam=NAC | NC100_ARATH NAC domain-containing protein 100 OS=Arabidopsis thaliana GN=NAC100 PE=2 SV=1 |
| Pm024898_0 | C2C2-Dof | transcription factor | -- | DOF33_ARATH Dof zinc finger protein DOF3.3 OS=Arabidopsis thaliana GN=DOF3.3 PE=1 SV=2 |
| Pm024912_0 | MYB | transcription factor | http://planttfdb.cbi.pku.edu.cn/family.php?fam=MYB | MYB06_ANTMA Myb-related protein 306 OS=Antirrhinum majus GN=MYB306 PE=2 SV=1 |
| Pm025009_0 | bHLH | transcription factor | http://planttfdb.cbi.pku.edu.cn/family.php?fam=bHLH | BIM1_ARATH Transcription factor BIM1 OS=Arabidopsis thaliana GN=BIM1 PE=1 SV=2 |
| Pm025026_0 | MADS | transcription factor | -- | AGL8_SOLTU Agamous-like MADS-box protein AGL8 homolog OS=Solanum tuberosum PE=2 SV=1 |
| Pm025253_0 | MYB | transcription factor | http://planttfdb.cbi.pku.edu.cn/family.php?fam=MYB | MYBG_DICDI Myb-like protein G OS=Dictyosteliumdiscoideum GN=mybG PE=3 SV=1 |
| Pm025274_0 | C2H2 | transcription factor | http://planttfdb.cbi.pku.edu.cn/family.php?fam=C2H2 | NUC_ARATH Zinc finger protein NUTCRACKER OS=Arabidopsis thaliana GN=NUC PE=2 SV=1 |
| Pm025830_0 | bHLH | transcription factor | http://planttfdb.cbi.pku.edu.cn/family.php?fam=bHLH | BH060_ARATH Transcription factor bHLH60 OS=Arabidopsis thaliana GN=BHLH60 PE=2 SV=1 |
| Pm025875_0 | C3H | transcription factor | http://planttfdb.cbi.pku.edu.cn/family.php?fam=C3H | C3H30_ARATH Zinc finger CCCH domain-containing protein 30 OS=Arabidopsis thaliana GN=At2g41900 PE=1 SV=2 |
| Pm025933_0 | HB | transcription factor | -- | HAT14_ARATH Homeobox-leucine zipper protein HAT14 OS=Arabidopsis thaliana GN=HAT14 PE=2 SV=3 |
| Pm026023_0 | HB | transcription factor | -- | BLH1_ARATH BEL1-like homeodomain protein 1 OS=Arabidopsis thaliana GN=BLH1 PE=1 SV=1 |
| Pm026268_0 | Trihelix | transcription factor | http://planttfdb.cbi.pku.edu.cn/family.php?fam=Trihelix | -- |
| Pm026327_0 | C2H2 | transcription factor | http://planttfdb.cbi.pku.edu.cn/family.php?fam=C2H2 | ZFP1_WHEAT Zinc finger protein 1 OS=Triticum aestivum PE=2 SV=1 |
| Pm026371_0 | FHA | transcription factor | -- | ABA2_PRUAR Zeaxanthin epoxidase, chloroplastic OS=Prunus armeniaca PE=2 SV=1 |
| Pm026389_0 | bHLH | transcription factor | http://planttfdb.cbi.pku.edu.cn/family.php?fam=bHLH | SPT_ARATH Transcription factor SPATULA OS=Arabidopsis thaliana GN=SPT PE=1 SV=1 |
| Pm026513_0 | ABI3VP1 | transcription factor | -- | VRN1_ARATH B3 domain-containing transcription factor VRN1 OS=Arabidopsis thaliana GN=VRN1 PE=2 SV=1 |
| Pm026571_0 | BES1 | transcription factor | http://planttfdb.cbi.pku.edu.cn/family.php?fam=BES1 | BEH2_ARATH BES1/BZR1 homolog protein 2 OS=Arabidopsis thaliana GN=BEH2 PE=1 SV=1 |
| Pm026593_0 | C2H2 | transcription factor | http://planttfdb.cbi.pku.edu.cn/family.php?fam=C2H2 | MGP_ARATH Zinc finger protein MAGPIE OS=Arabidopsis thaliana GN=MGP PE=1 SV=1 |
| Pm026635_0 | MYB | transcription factor | http://planttfdb.cbi.pku.edu.cn/family.php?fam=MYB | -- |
| Pm026679_0 | bZIP | transcription factor | http://planttfdb.cbi.pku.edu.cn/family.php?fam=bZIP | OCS1_MAIZE Ocs element-binding factor 1 OS=Zea mays GN=OBF1 PE=2 SV=2 |
| Pm026729_0 | Orphans | transcriptional regulator | -- | STHY_ARATH Probable salt tolerance-like protein At1g78600 OS=Arabidopsis thaliana GN=At1g78600 PE=2 SV=2 |
| Pm026740_0 | C3H | transcription factor | http://planttfdb.cbi.pku.edu.cn/family.php?fam=C3H | C3H30_ORYSJ Zinc finger CCCH domain-containing protein 30 OS=Oryza sativa subsp. japonica GN=Os04g0663200 PE=2 SV=3 |
| Pm026979_0 | Tify | transcription factor | -- | TIF3B_ARATH Protein TIFY 3B OS=Arabidopsis thaliana GN=TIFY3B PE=1 SV=1 |
| Pm027048_0 | MYB | transcription factor | http://planttfdb.cbi.pku.edu.cn/family.php?fam=MYB | MYBC_MAIZE Anthocyanin regulatory C1 protein OS=Zea mays GN=C1 PE=2 SV=1 |
| Pm027113_0 | Tify | transcription factor | -- | TI10A_ARATH Protein TIFY 10A OS=Arabidopsis thaliana GN=TIFY10A PE=1 SV=1 |
| Pm027119_0 | ARF | transcription factor | http://planttfdb.cbi.pku.edu.cn/family.php?fam=ARF | ARFS_ARATH Auxin response factor 19 OS=Arabidopsis thaliana GN=ARF19 PE=1 SV=2 |
| Pm027273_0 | C3H | transcription factor | http://planttfdb.cbi.pku.edu.cn/family.php?fam=C3H | C3H20_ARATH Zinc finger CCCH domain-containing protein 20 OS=Arabidopsis thaliana GN=At2g19810 PE=2 SV=1 |
| Pm027402_0 | MBF1 | transcriptional regulator | -- | MBF1C_ARATH Multiprotein-bridging factor 1c OS=Arabidopsis thaliana GN=MBF1C PE=1 SV=1 |
| Pm027421_0 | HSF | transcription factor | http://planttfdb.cbi.pku.edu.cn/family.php?fam=HSF | HSFC1_ARATH Heat stress transcription factor C-1 OS=Arabidopsis thaliana GN=HSFC1 PE=2 SV=1 |
| Pm027462_0 | LUG | transcriptional regulator | -- | LEUNG_ARATH Transcriptional corepressor LEUNIG OS=Arabidopsis thaliana GN=LUG PE=1 SV=2 |
| Pm027468_2 | GRAS | transcription factor | http://planttfdb.cbi.pku.edu.cn/family.php?fam=GRAS | GAI_ARATH DELLA protein GAI OS=Arabidopsis thaliana GN=GAI PE=1 SV=1 |
| Pm027559_0 | C2C2-YABBY | transcription factor | -- | YAB5_ARATH Axial regulator YABBY 5 OS=Arabidopsis thaliana GN=YAB5 PE=2 SV=1 |
| Pm027656_0 | HB | transcription factor | -- | KNAT3_ARATH Homeobox protein knotted-1-like 3 OS=Arabidopsis thaliana GN=KNAT3 PE=1 SV=1 |
| Pm028024_0 | OFP | transcription factor | -- | -- |
| Pm028504_0 | Pseudo ARR-B | transcriptional regulator | -- | PRR95_ORYSJ Two-component response regulator-like PRR95 OS=Oryza sativa subsp. japonica GN=PRR95 PE=2 SV=1 |
| Pm028731_0 | MYB | transcription factor | http://planttfdb.cbi.pku.edu.cn/family.php?fam=MYB | LHY_ARATH Protein LHY OS=Arabidopsis thaliana GN=LHY PE=1 SV=2 |
| Pm028732_0 | MYB | transcription factor | http://planttfdb.cbi.pku.edu.cn/family.php?fam=MYB | LHY_ARATH Protein LHY OS=Arabidopsis thaliana GN=LHY PE=1 SV=2 |
| Pm029015_0 | AP2-EREBP | transcription factor | -- | RAVL1_ARATH AP2/ERF and B3 domain-containing transcription repressor TEM1 OS=Arabidopsis thaliana GN=TEM1 PE=1 SV=1 |
| Pm029019_0 | G2-like | transcription factor | http://planttfdb.cbi.pku.edu.cn/family.php?fam=G2-like | ARR18_ARATH Two-component response regulator ARR18 OS=Arabidopsis thaliana GN=ARR18 PE=2 SV=2 |
| Pm029052_0 | AP2-EREBP | transcription factor | -- | EF118_ARATH Ethylene-responsive transcription factor ERF118 OS=Arabidopsis thaliana GN=ERF118 PE=2 SV=1 |
| Pm029117_0 | AUX/IAA | transcriptional regulator | -- | IAA34_ARATH Auxin-responsive protein IAA34 OS=Arabidopsis thaliana GN=IAA34 PE=2 SV=1 |
| Pm029185_0 | MYB | transcription factor | http://planttfdb.cbi.pku.edu.cn/family.php?fam=MYB | MYB4_ARATH Transcription repressor MYB4 OS=Arabidopsis thaliana GN=MYB4 PE=1 SV=1 |
| Pm029235_0 | G2-like | transcription factor | http://planttfdb.cbi.pku.edu.cn/family.php?fam=G2-like | KAN2_ARATH Probable transcription factor KAN2 OS=Arabidopsis thaliana GN=KAN2 PE=2 SV=1 |
| Pm029437_0 | MYB | transcription factor | http://planttfdb.cbi.pku.edu.cn/family.php?fam=MYB | MY113_ARATH Transcription factor MYB113 OS=Arabidopsis thaliana GN=MYB113 PE=1 SV=1 |
| Pm029438_0 | MYB | transcription factor | http://planttfdb.cbi.pku.edu.cn/family.php?fam=MYB | MY113_ARATH Transcription factor MYB113 OS=Arabidopsis thaliana GN=MYB113 PE=1 SV=1 |
| Pm029440_0 | MYB | transcription factor | http://planttfdb.cbi.pku.edu.cn/family.php?fam=MYB | MY113_ARATH Transcription factor MYB113 OS=Arabidopsis thaliana GN=MYB113 PE=1 SV=1 |
| Pm029442_0 | MYB | transcription factor | http://planttfdb.cbi.pku.edu.cn/family.php?fam=MYB | MY113_ARATH Transcription factor MYB113 OS=Arabidopsis thaliana GN=MYB113 PE=1 SV=1 |
| Pm029489_0 | MYB | transcription factor | http://planttfdb.cbi.pku.edu.cn/family.php?fam=MYB | MYBC_MAIZE Anthocyanin regulatory C1 protein OS=Zea mays GN=C1 PE=2 SV=1 |
| Pm029646_0 | SNF2 | transcriptional regulator | -- | INO80_ARATH DNA helicase INO80 complex homolog 1 OS=Arabidopsis thaliana GN=INO80 PE=2 SV=2 |
| Pm029981_0 | Orphans | transcriptional regulator | -- | ARR3_ARATH Two-component response regulator ARR3 OS=Arabidopsis thaliana GN=ARR3 PE=1 SV=1 |
| Pm030099_0 | NAC | transcription factor | http://planttfdb.cbi.pku.edu.cn/family.php?fam=NAC | NAC29_ARATH NAC domain-containing protein 29 OS=Arabidopsis thaliana GN=NAC029 PE=2 SV=1 |
| Pm030137_0 | Sigma70-like | transcription factor | -- | RPOD_NOSS1 RNA polymerase sigma factor rpoD OS=Nostoc sp. (strain PCC 7120 / UTEX 2576) GN=rpoD PE=3 SV=1 |
| Pm030138_0 | TCP | transcription factor | http://planttfdb.cbi.pku.edu.cn/family.php?fam=TCP | TCP11_ARATH Transcription factor TCP11 OS=Arabidopsis thaliana GN=TCP11 PE=2 SV=1 |
| Pm030172_0 | AP2-EREBP | transcription factor | -- | AP2_ARATH Floral homeotic protein APETALA 2 OS=Arabidopsis thaliana GN=AP2 PE=1 SV=1 |
| Pm030235_0 | AP2-EREBP | transcription factor | -- | RA211_ARATH Ethylene-responsive transcription factor RAP2-11 OS=Arabidopsis thaliana GN=RAP2-11 PE=2 SV=1 |
| Pm030475_0 | AP2-EREBP | transcription factor | -- | WIN1_ARATH Ethylene-responsive transcription factor WIN1 OS=Arabidopsis thaliana GN=WIN1 PE=2 SV=1 |
| Pm031033_0 | OFP | transcription factor | -- | -- |
| Pm031086_0 | Orphans | transcriptional regulator | -- | ARR9_ARATH Two-component response regulator ARR9 OS=Arabidopsis thaliana GN=ARR9 PE=1 SV=1 |
| Pm031275_0 | ARF | transcription factor | http://planttfdb.cbi.pku.edu.cn/family.php?fam=ARF | ARFR_ORYSJ Auxin response factor 18 OS=Oryza sativa subsp. japonica GN=ARF18 PE=2 SV=1 |
| Pm031349_0 | ARF | transcription factor | http://planttfdb.cbi.pku.edu.cn/family.php?fam=ARF | ARFC_ARATH Auxin response factor 3 OS=Arabidopsis thaliana GN=ARF3 PE=1 SV=2 |
| Pm031356_0 | VOZ | transcription factor | http://planttfdb.cbi.pku.edu.cn/family.php?fam=VOZ | -- |
